# Temporal judgments of actions following unilateral brain damage

**DOI:** 10.1038/s41598-022-26070-9

**Published:** 2022-12-15

**Authors:** Valentina Pacella, M. Scandola, M. Bà, N. Smania, M. Beccherle, E. Rossato, D. Volpe, Valentina Moro

**Affiliations:** 1 Groupe d’Imagerie NeurofonctionnelleInstitut des Maladies Neurodégénératives-UMR 5293, CNRS, CEA, University of Bordeaux, 146 Rue Léo Saignat, CS 61292, 33076 Bordeaux Cedex, France; 2 Brain Connectivity and Behaviour Laboratory, Sorbonne Universities, Paris, France; 3 NPSY-Lab.VR, Department of Human Sciences, University of Verona, Lungadige Porta Vittoria 17, 37129 Verona, Italy; 4 Neurorehabilitation Unit, Department of Neurosciences, Hospital Trust of Verona, Verona, Italy; 5 Department of Psychology, University La Sapienza, Rome, Italy; 6 Department of Rehabilitation, IRCSS Sacro Cuore Don Calabria, 37024 Negrar, Verona, Italy; 7 Department of Neurorehabilitation, Parkinson’s Disease Excellence Center, Fresco Institute Italy - NYU Langone, Casa di Cura Villa Margherita via Costacolonna n 1 Arcugnano, Vicenza, Italy

**Keywords:** Stroke, Neuroscience

## Abstract

Sense of time is a complex construct, and its neural correlates remain to date in most part unknown. To complicate the frame, physical attributes of the stimulus, such as its intensity or movement, influence temporal perception. Although previous studies have shown that time perception can be compromised after a brain lesion, the evidence on the role of the left and right hemispheres are meager. In two experiments, the study explores the ability of temporal estimation of multi-second actions and non-biological movements in 33 patients suffering from unilateral brain lesion. Furthermore, the modulatory role of induced embodiment processes is investigated. The results reveal a joint contribution of the two hemispheres depending not only on different durations but also on the presence of actions. Indeed, the left hemisphere damaged patients find it difficult to estimate 4500 ms or longer durations, while the right hemisphere damaged patients fail in 3000 ms durations. Furthermore, the former fail when a biological action is shown, while the latter fail in non-biological movement. Embodiment processes have a modulatory effect only after right hemisphere lesions. Among neuropsychological variables, only spatial neglect influences estimation of non-biological movement.

## Introduction

Time is a crucial dimension of human life, and discussions on the nature of time have always been prominent in scientific and philosophical debates (e.g.^1,2^). However, the “sense of time” is multifaceted, as it involves different abilities such as time estimation, discrimination among durations, reproduction of time intervals, synchronization with a rhythm and so on. Furthermore, time perception is influenced by multiple factors, such as the emotional valence of a stimulus^3^, the presence of a stressful environment^4^, depression or anxiety^5–7^. Ultimately, sense of time represents a subjective, internal phenomenon, not objectively representable or verbally identifiable (“the subjective experience of time”^8,9^) and thus difficult to study.

Experimental and clinical investigations indicate a close relationship between body and time perception. On one side, body impacts on time discrimination, as shown by the correlation between interoception (i.e. the capacity of perceiving information coming from the internal body and to be aware of these information) and accuracy in estimation of time intervals^10^, or by the modulation of embodiment processes in the judgement of time intervals^11,12^. On the other hand, the embodiment of external objects are facilitated by the temporal synchronicity of visuo-tactile stimuli, as shown by the well-known paradigm of hand and full body illusions^13–15^. The intensity of movements influences time perception as well. For example, the elapsing time of the observation of dancing statues in motion is estimated longer or shorter according to the intensity of the movement represented^12^. Furthermore, timing hinges on brain structures typically involved with motor control, and timing is largely informed by motor related signals^16–18^.

Although the neural mechanisms underlying time perception are still matter of investigation^19^, previous studies suggest that time is processed in different manners depending on the duration of the time intervals and the typology of the tasks employed in the experimental paradigms^20,21^. For example, the regulation of circadian cycles (with a 24-h scale) involves subcortical structures, in particular the suprachiasmatic nucleus of the hypothalamus, while very fast processes on milli-second timing scales are strongly associated with cerebellar and motor systems^22^. Multi-second timing is probably underpinned by a thalamo-cortico-striatal network, involving in particular the striatum, the thalamus, the prefrontal and supplementary motor areas and the parietal cortices^23–27^. The insular cortex has been found to be involved in time processing as well, in particular in the reproduction of intervals (with supra-second intervals^28^), in the retrospective judgement of time elapsing^9^ and explicit time prediction^29^. Finally, the medial temporal cortex and hippocampus contribute to time representations, in particular when sequential processing is asked and independently from the duration range of intervals^30–32^.

The thalamo-cortico-striatal network, including frontal, parietal, insular cortices and cerebellum supports one of the most influential hypotheses of time processing, namely *the internal clock model*, that considers time as the result of the accumulation of pulses from a neural pacemaker^33–35^. Specifically, the model suggests that time intervals perception is built via three consequential steps^36^: (i) a clock stage, during which a stimulus or event is converted by a neural pacemaker in timing signals (or pulses) that are collected by an accumulator (thanks to attentional processes); (ii) a step where these accumulated signals are stored in working memory and quantified to be (iii) compared with previously stored values for that stimulus duration. From this comparison and when the two values match closely enough, a decision on duration is taken. The right insula has been suggested as the temporal accumulator, which is then associated with the prefrontal cortex and cerebellum to produce motor responses^37^. Furthermore, striatal and cerebellum activity might be mainly involved in the stage of converting timing signals in neural information, as these two structures result to be crucial in beat-count tasks and synchronization processes for the planning of decision making^24,38^.

Synchronization is at the centrum of another model for time perception, the *beat frequency model of timing*^24,39–41^, that advances the hypothesis that time is coded by the coincidental activation of striatal spiny neurons by cortical neural oscillators^42,43^.

The role of the mesocortical pathway in timing mechanisms is hypothesised in the *hybrid model of time processing*^44,45^ as well. The model advances the theory that a main core timing mechanism relies on the mesocortical pathway and interacts with domain-dependent brain structures. Thus, different structures are engaged in time processing according to the different task/stimulus modalities.

In this complex frame, the contribution from neurological patients is potentially relevant in the understanding of time processing, both in terms of identification of the neural correlates and for a better theoretical comprehension of the cognitive processes of temporal judgments. However, data are still meagre and inconsistent. It is known that subjective experience of time tends to be less efficient after a brain lesion and that the sense of malaise correlates with errors in time estimation^46,47^. However, the potential differences between left and right hemisphere damaged patients are to date in most part unexplored.

Underestimation of time intervals has been found in right hemisphere damaged patients, with worse performance in the presence of spatial disorders such as spatial neglect^48–51^ (but see^9^ who do not find any differences in right brain damaged- RBD-with and without neglect). Only a few studies compare patients suffering from left and right hemisphere lesions, with inconsistent results^9,47,52–54^. Indeed, while some studies show the right but not left hemisphere damaged patients underestimating time intervals^52,53^, a more recent study^47^ reveals that symptoms may vary based on the time from the lesion onset. In the acute post-stroke phase and in transient ischemic attack patients, time underestimation is found following both left and right damages, while three months later, the disorder persists only in left-side stroke and in right side with (but not without) neglect. In these latter, a predictive role of cognitive impairment in short-term and working memory abilities is also reported^47^. Some specificities have also been found between the prospective and retrospective time estimation, however without differences associated with the side of lesion^9^.

What at the best of our knowledge remains unexplored is the role of the body in temporal judgements of actions. This study aims to fill this gap and respond to the following questions: (i) does a hemispheric specialization exist in time processing? and (ii) is the duration of actions processed in a different way with respect to duration of other typologies of stimuli (i.e. non-biological movement)? If so, (iii) is temporal judgement of actions associated with other cognitive functions (i.e. the presence of limb apraxia, spatial or attentional disorders, awareness of motor deficits)? Finally, (iv) if the time of action is something special for our brain, can the embodiment of actions seen have modulatory effects?

We advanced the hypothesis that two syndromes might impact on temporal estimation of action, namely limb apraxia and anosognosia for hemiplegia, that are usually associated with left and right hemisphere damage, respectively. Limb apraxia is the inability to perform specific and predefined actions^55^, as a consequence of lesions involving fronto-parietal networks^56–58^. Anosognosia for hemiplegia (i.e. the lack of knowledge of one own’s motor deficits^59^) is usually reported after right brain damages as a consequence of lesions in structures which are also involved in time processing, such as the insula^60^ the fronto-parietal network (with the superior longitudinal fasciculus) and the fronto-striatal network^61–63^. Crucially for the hypotheses of this study, in both these two clinical conditions, processes of embodiment induced through the mirror box illusion^64^ or self-observation in a video^65,66^ are efficacious in modulating patients’ symptoms.

A group of 33 brain damaged patients with unilateral lesions participated in a task of estimation of various durations of actions seen in a screen. A group of healthy subjects matched for age, gender and education served as control. As a no-body condition, the same durations were used during the presentation of a non-biological movement of a little ball while moving vertically toward a target.

In the presence of a hemispheric lateralization of duration estimation, we expected that the two groups perform in a different way when compared to each other and to the control group in both the tasks. If, on the contrary, differences were found only in the action condition, this would be interpreted as an index of the presence of specific processes devoted to action durations. In this case, we also expected that action-related functions, such as motor deficits, apraxia and anosognosia for hemiplegia, impact time processing. This category specific deficit (e.g. for action stimuli) might be present in one group of patients and not the other. Finally, in the case of a role of body representations in temporal estimation of actions, we expected that the experimental induction of the embodiment of the arm that performs the actions modulates patients’ performance.

In the study, these hypotheses have been tested by means of two sequential experiments: (i) the Action Time Estimation (ATE), with, as a control task without actions, the Movement Time Estimation (MTE) and (ii) the Action Time Estimation with manipulation of Embodiment (ATE-embodiment and ATE-no hand).

## Results

20 right brain damaged (RBD) and 13 left brain damaged (LBD) patients participated in the study. All the patients performed Experiment 1, while a subgroup (n.16) participated also in Experiment 2 (11 RBD, 5 LBD). 42 healthy participants served as control group (C).

A neuropsychological assessment (Table 1) investigated: general cognitive functions (Mini Mental State Examination^67^, MMSE); language comprehension (Aachener Aphasia Test^68^, AAT); ideomotor apraxia^69^; awareness for contralesional upper limb motor impairment^70,71^; short term memory (digit span forward^72^); spatial attention deficits^73^; and personal neglect^74^. To exclude the presence of general cognitive impairment, the MMSE was administered to the control group as well. As the assessment for motor unawareness identified 7 anosognosic patients (AHP) in the right brain damaged group, these patients were separated into two groups, with a total of four groups in both Experiment 1 (AHP = 7; RBD = 13; LBD = 13, C = 42) and Experiment 2 (AHP = 4; RBD = 7; LBD = 5, C = 16). The results of the neuropsychological assessment are shown in Table 1. AHP patients have worse performance than the other groups not only in the task assessing awareness (Visual-Analogue Test for Anosognosia for Motor Impairment, VATA-m), but also in general cognitive functions, oral comprehension and spatial attention. Consistently with expectations, RBD patients (in Experiment 1) presented with a significant spatial attention deficit compared to the LBD group, while the LBD group (in Experiment 2) show significantly higher scores (i.e. more errors) in the test for apraxia when compared to the RBD group (see Supplementary Materials, SM1 for the statistical results).

**Table 1:** Mean and ± standard deviation of demographic, clinical and neuropsychological assessments are shown for the three groups of patients (RBD, AHP, LBD) and the healthy controls group.

	RBD N = 13	AHP N = 7	LBD N = 13		Controls N = 42
Age	67.62 ± 10.63	70.43 ± 11.65	64.84 ± 15.25		63.86 ± 11.17
Education	10.54 ± 4.7	11.14 ± 4.38	10.46 ± 4.18		10.98 ± 4.38
Interval (days)	116.62 ± 160.91	101.67 ± 59.2	70.46 ± 85.25		
Motricity index (MRC-UL)	11 ± 15.31	6.86 ± 12.21	18.38 ± 17.22		
EHI	88.08 ± 15.75	85.71 ± 18.12	78.08 ± 25.21		82.31 ± 35.7
MMSE	26.36 ± 2.17	22.04 ± 2.67^▲^	20.51 ± 9.54		27.83 ± 1.36
Token test (err.)	1.54 ± 1.98	4.71 ± 1.11^▲■^	1.85 ± 2.25		
Oral comprehension	26.15 ± 3.76	22.43 ± 3.09^■^	23.77 ± 10.01		
Comprehension of written sentences	26.15 ± 3.76	24.57 ± 2.44	26 ± 3.74		
Ideomotor apraxia (err.)	0.12 ± 0.3	0.21 ± 0.39	0.54 ± 1.27		
Digit span	4.68 ± 1.31	4.77 ± 0.77	4.12 ± 1.57		
Comb test	− 0.01 ± 0.26	− 0.03 ± 0.35	0.08 ± 0.21		
Line crossing	33.15 ± 5.97	27.71 ± 10.08^■^	35.85 ± 0.38		
Line bisection	6.08 ± 2.63^■^	3.71 ± 3.04^■^	8.62 ± 0.87		
VATA-m (UL)	0.39 ± 4.59	16 ± 1.53^▲■^	0 ± 1.68		
Bisiach score	0	1.5 ± 0.77	0		

^▲^Significant differences of a group (AHP or LBD) with respect to the RBD group. ^■^Significant differences of a group (AHP or RBD) with respect to the LBD group. Only the MMSE was administered to participants in the control group, in order to exclude any signs of mental deterioration. RBD, Right brain damaged patients; AHP, patients suffering from Anosognosia for Hemiplegia; LBD, left brain damaged patients; MRC-UL, Medical Research Council-Upper Limb; EHI, Edinburgh Handedness Inventory; MMSE, Mini Mental State Examination (cut-off = 23.8); err., number of error; Token test, severe deficit = > 40 err.; Oral comprehension, severe deficit = score < 43; Comprehension of written sentences = score < 43; Ideomotor apraxia = not validated, clinical assessment; Digit span = 3; Comb and razor = < − 0.11; Line crossing = ≥ 34 ; Line bisection = ≥ 7; VATA-m, Visual-Analogue Test for Anosognosia for Motor Impairment (cut-offs > 6.2); UL, upper limb; Bisiach score = 1.5.

### Experiment 1: Action time estimation task (ATE)

**Figure 1:**
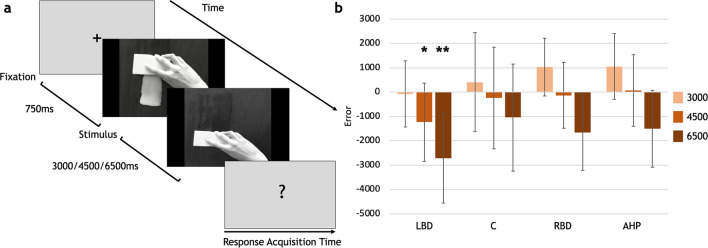
(**a**) Flow of events in the ATE task. There were no response time constraints and response time acquisition lasted until the patient's response. (**b**) Error differences between controls and each patient group. *p < 0.05; **p < 0.001. Boxes represent the mean error of each group, for each duration; bars represent standard deviation. LBD, left brain-damaged patients; C, controls; RBD, right brain-damaged patients; AHP, anosognosia for hemiplegia patients.

**Figure 2:**
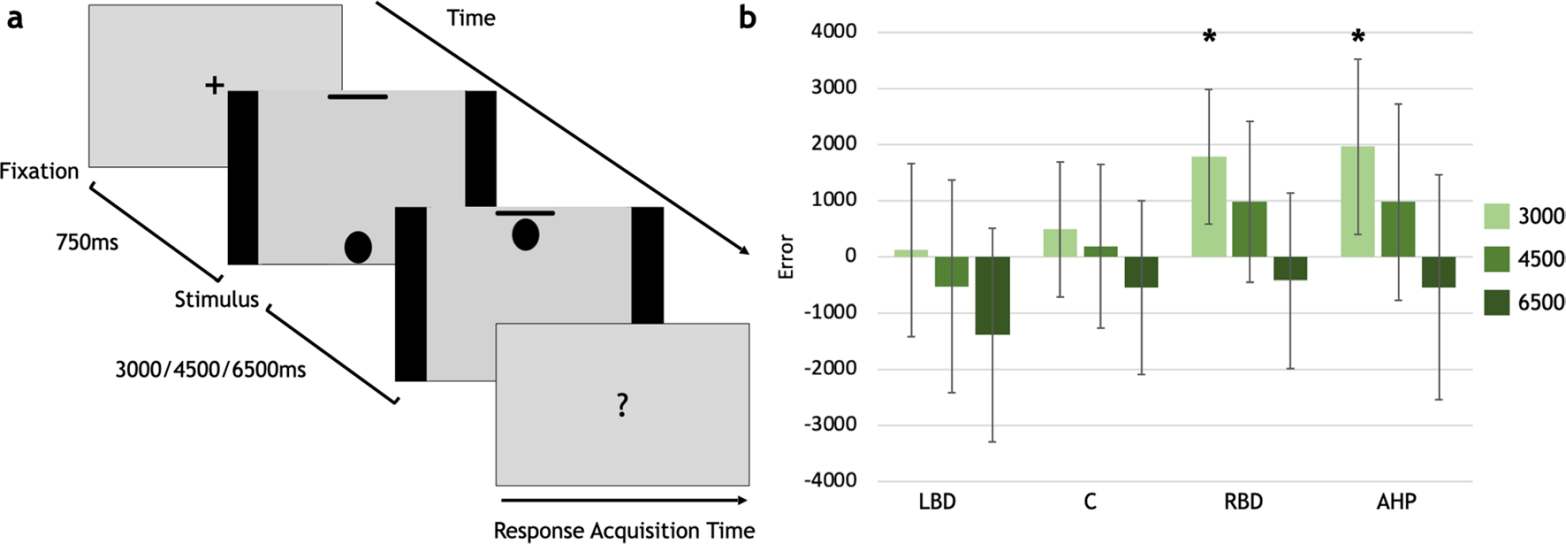
(**a**) Flow of events in the MTE task. As in the ATE task, there were no response time constraints and response time acquisition lasted until the patient's response. (**b**) Error differences between controls and each patient group. *p < 0.05. Boxes represent the mean error of each group, for each duration; bars represent standard deviation. LBD, left brain-damaged patients; C, controls; RBD, right brain-damaged patients; AHP, anosognosia for hemiplegia patients.

The Action Time Estimation (ATE) task was used to measure potential differences among the groups in estimating the temporal durations (i.e. 3000 ms, 4500 ms, 6500 ms) of actions that were presented on a screen by means of a series of videos. These included the use of a tool (by the left or right hand) with actions being seen from a first-person perspective and performed in a vertical trajectory (Fig. 1a). In a control task, a vertical movement of a circle toward a horizontal line (Movement-Time Estimation MTE) was shown in the videos (Fig. 2a).

The raw values of estimates indicate that differences in the various durations (i.e. 3000 ms, 4500 ms, 6500 ms) were identified by all the four groups of participants (SM2 for details), and the analyses were conducted separately for each duration. To quantify the errors in the estimation of durations in both ATE and MTE tasks, an index was computed for each response as the difference between the participant’s subjective estimation and the actual duration of action/movement. In this way, a score close to zero indicates accurate estimation, a score above zero shows overestimation, and a score below zero indicates underestimation of the duration.

Furthermore, a preliminary analysis of responses in the ATE indicated the absence of differences between ipsilesional and contralesional hands in the three groups of patients and between right and left hands in controls (Fig. SM2). Thus, the responses referred to left and right hand videos were collapsed in a unique score.

For each duration, a pairwise *t*-test compared the performance of the control group with the performance of each of the three patient groups. Bonferroni correction was applied to control for multiple comparisons. The comparison between the LBD and controls’ errors shows significant underestimation of the durations in the LBD, for the durations of 4500 ms (*p* = 0.02) and 6500 ms (*p* < 0.001) (Fig. 1b).The responses of the AHP and RBD groups did not differ from controls in any of the three durations (AHP v. C, 3000 ms: *p* = 0.14; 4500 ms: *p* = 0.59; 6500 ms: *p* = 0.42; RBD v. C = 3000 ms *p* = 0.15; 4500 ms *p* = 0.85; 6500 ms *p* = 0.18; Fig. 1b).

### Movement time estimation (MTE)

The AHP group significantly overestimated only the durations of video lasting 3000 ms (*p* = 0.003) (AHP v. C: 4500 ms *p* = 0.08; 6500 ms *p* = 0.59; Fig. 2), as well as the RBD group who significantly overestimated the 3000 ms duration when compared to controls (*p* = 0.004) (RBD v. C = 4500 ms: *p* = 0.49; 6500 ms: *p* = 0.73). The LBD group errors did not differ from controls (LBD v. C = 3000 ms: *p* = 0.5; 4500 ms: *p* = 0.21; 6500 ms: *p* = 0.13).

### Explorative analyses of lesions

**Figure 3:**
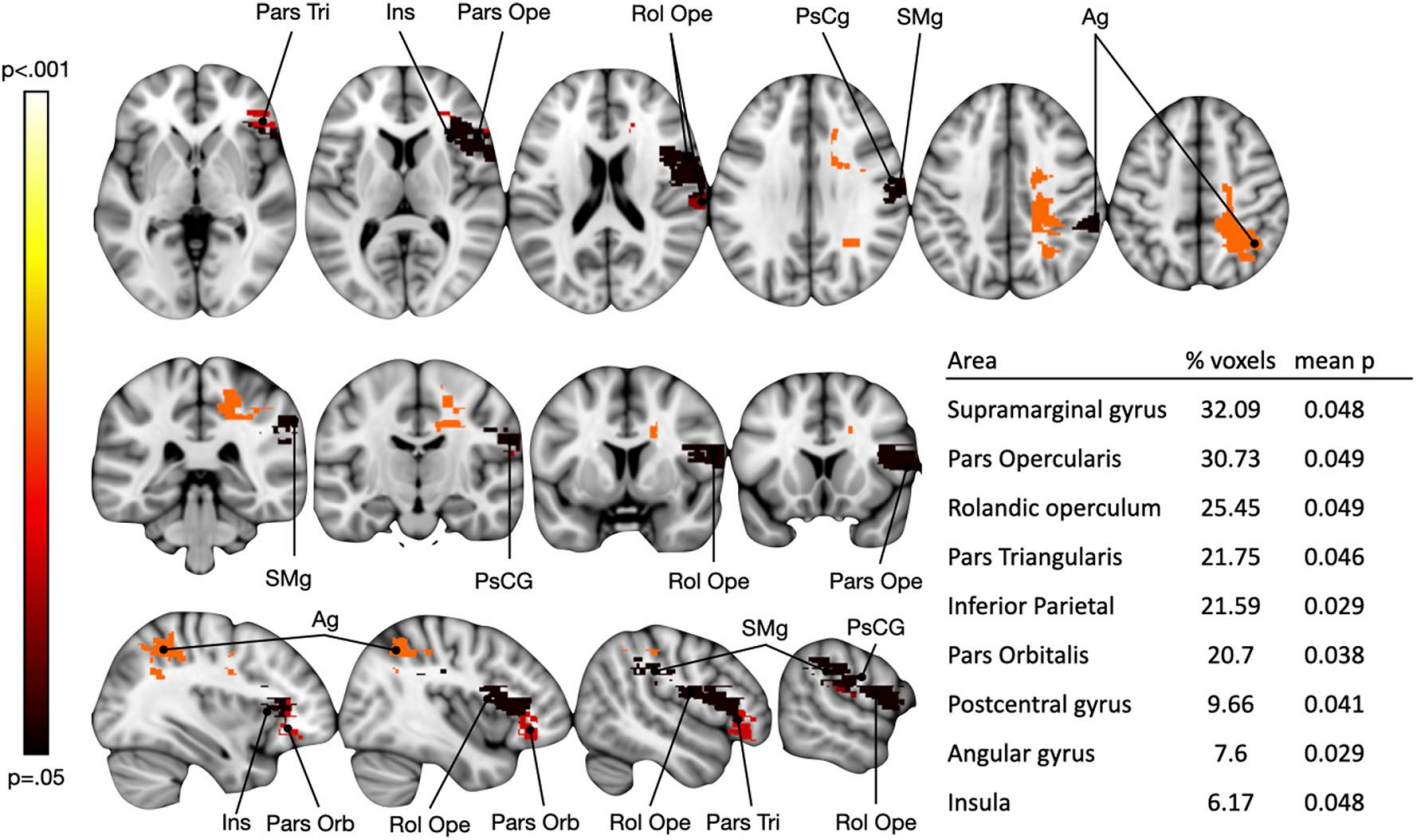
The results from the lesion analysis computed on the errors in temporal estimation in the MTE (3000 ms duration) for the RBD + AHP groups (9 patients in total). Voxels with *p* < 0.05 FDR-corrected are represented. The table shows the percentage of voxels (%voxels) and the mean *p* value for each areas that resulted to be associated with errors in the 3000 ms-MTE task. Ag, angular gyrus; Ins, insular cortex; Pars Orb, pars orbitalis of the inferior frontal gyrus; Pars Ope, pars opercularis of the inferior frontal gyrus; Pars tri, pars triangularis of the inferior frontal gyrus; PsCg, postcentral gyrus; Rol Ope, rolandic operculum; SMg, supramarginal gyrus.

Given the limited number of patients whose neuroimaging data were available, an explorative lesion analysis was performed to investigate the neural correlates of errors in estimation of durations (differences in the sense of underestimation were transformed into positive scores). The analysis was done only for RBD (N images = 5) and AHP patients (N images = 4), which were considered as a whole group, for the scores in the 3000 ms duration (i.e. the only duration where a difference is present with respect to controls, in MTE task). Unfortunately, missing data for LBD (N images available = 2) did not allow the same analysis for this group. The manually drawn lesions (described below) were used as dependent variables in a regression analysis, and the errors in the 3000 ms duration of the MTE task were used as independent variable. Threshold-Free Clusters Enhancement option was applied to boost cluster-like structures of voxels and results that survived 1000 permutations testing were controlled for family-wise error rate (*p* < 0.05). The involvement of the inferior parietal and frontal cortices, rolandic operculum, postcentral gyrus and insula in the overestimation of short durations is suggested by the results (Fig. 3).

### Experiment 2: Modulation from embodiment

**Figure 4:**
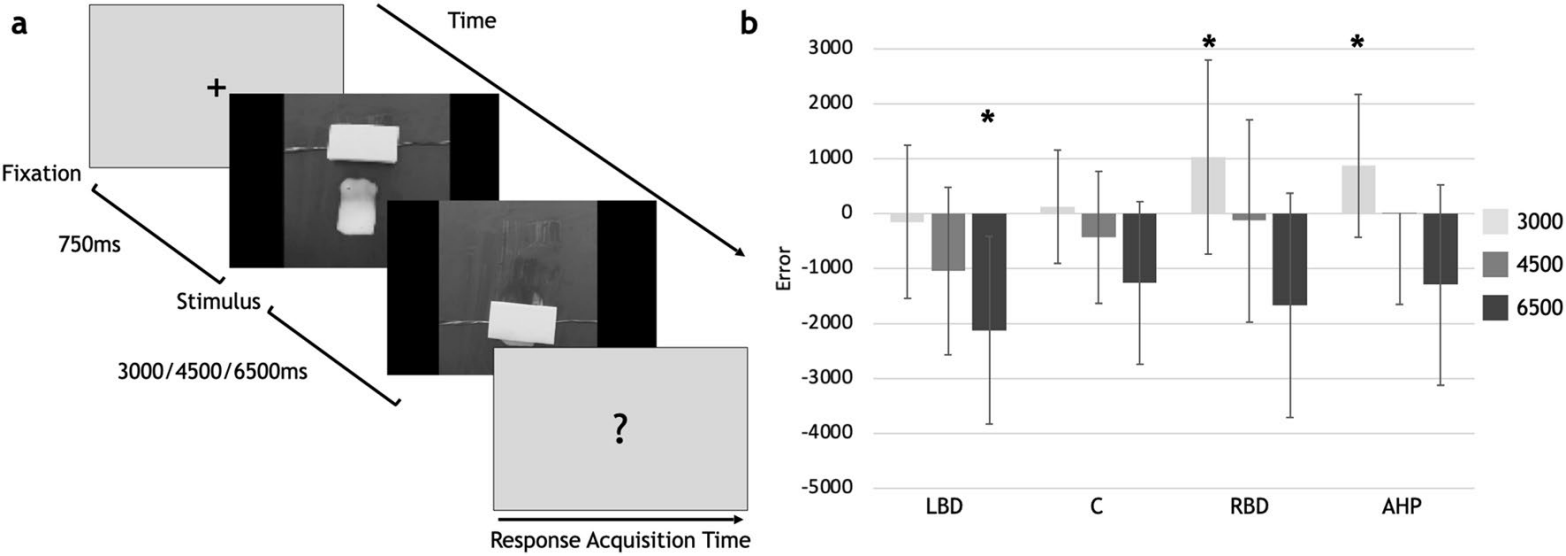
(**a**) Flow of events in the ATE-no hand task. As in the previous tasks, there were no response time constraints and response time acquisition lasted until the patient's response. (**b**) Error differences between controls and each patient group. *p < 0.05. Boxes represent the mean error of each group, for each duration; bars represent standard deviation. LBD, left brain-damaged patients; C, controls; RBD, right brain-damaged patients; AHP, anosognosia for hemiplegia patients.

**Figure 5:**
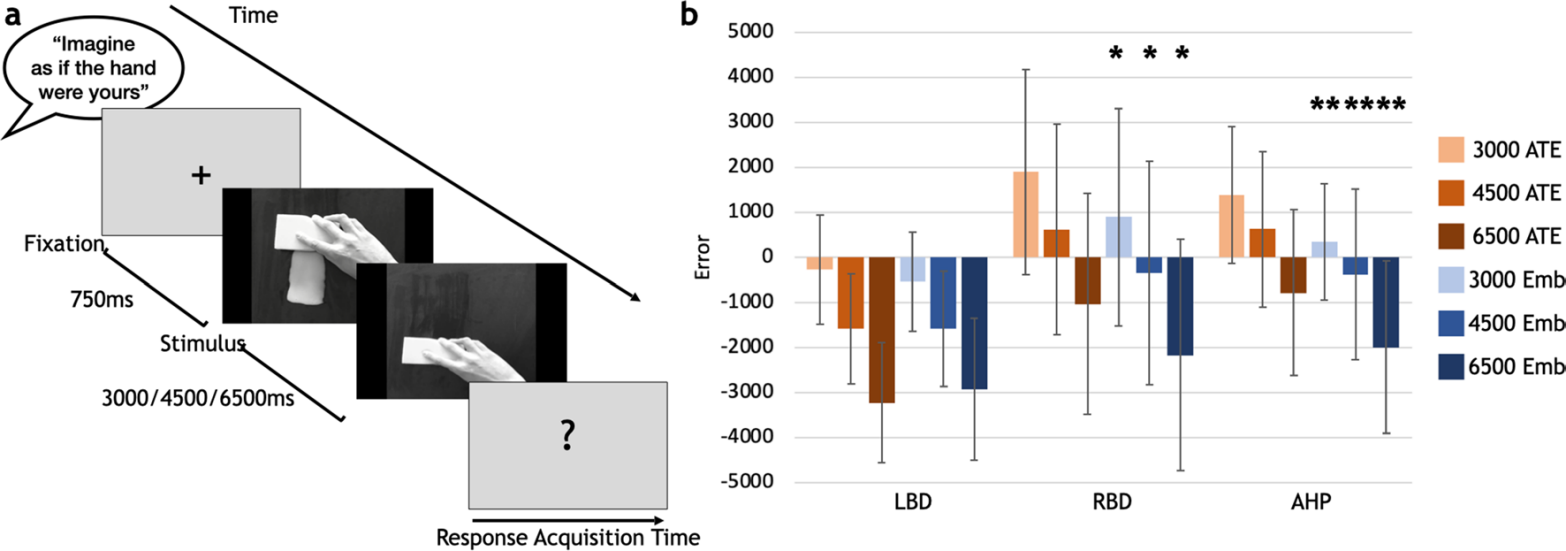
The figure represents (**a**) Flow of events in the ATE-embodiment task. There were no response time constraints and response time acquisition lasted until the patient's response. (**b**) Mean and standard deviation of the ATE (in orange shades) and ATE-Embodiment (in blue shades) for each group. *p < 0.05; **p < 0.01. The statistic refers to the comparison of errors performed in the ATE-embodiment versus ATE tasks, within each patients’ group. LBD, left brain-damaged patients; RBD, right brain-damaged patients; AHP, anosognosia for hemiplegia patients.

As a different pattern was found among the groups in the estimation of actions and non-biological movement, two new conditions were implemented in order to investigate the potential role of embodiment in judgments of action duration. In one condition, the embodiment was inhibited by removing the hand from the videos, such that actions did not include the presence of an effector (ATE-No Hand; Fig. 4a). In the other condition, the embodiment was artificially forced by the verbal instruction given during the task (see Material and methods for details and Fig. 5a). For each patient group, the estimation errors in ATE-no hand were compared to the performance in ATE task and to the control group.

In ATE-No Hand, RBD and AHP overestimated the shortest duration of 3000 ms (AHP v. C, 3000 ms: *p* = 0.035; 4500 ms: *p* = 0.3; 6500 ms: *p* = 0.85; RBD v. C = 3000 ms *p* = 0.015; 4500 ms *p* = 0.43; 6500 ms *p* = 0.37; Fig. 4), thus showing the same pattern as in MTE. LBD underestimated the 6500 ms actions (LBD v. C, 3000 ms: *p* = 0.38; 4500 ms: *p* = 0.06; 6500 ms: *p* = 0.03) but not the 4500 ms, as in ATE. A difference between ATE and ATE-No Hand was found only for the LBD group (SM3).

Induction of a forced embodiment (by means of specific instructions “Imagine as if the hand you are looking at was your hand”, see “Methods”) seems to impact the AHP and RBD groups’ performance. Indeed, in the ATE-Embodiment, the AHP group significantly underestimate the durations when compared to the ATE task for all the durations (3000 ms: t = 3.06; *p* = 0.007; 4500 ms: t = 2.22; *p* = 0.04; 6500 ms: t = 2.2; *p* = 0.041 Fig. 5b), with responses more precise (i.e. more close to the real durations for 3000 ms and 4500 ms). In the same way, the RBD group significantly underestimate the Hand-embodiment condition, compared to the Hand condition in all the durations (3000 ms: t = 3.47; *p* = 0.002; 4500 ms: t = 3.14; *p* = 0.005; 6500 ms: t = 3.01; *p* = 0.007 Fig. 2b). No differences have been found comparing the LBD group’s performance in the ATE and ATE-embodiment tasks. However, no difference has been found between each patient group and the controls in the ATE Embodiment task.

### Clinical correlations

Pearson’s correlations were performed (and corrected for Bonferroni multiple comparisons) between the errors of each task (and each duration) and the clinical variables that are different among the patients’ groups. No significant correlation was found (see SM4).

## Discussion

This study was aimed at answering various questions, all driven by a common issue, namely how time is estimated in our brain. In particular, the two experimental paradigms focused on the investigation of potential different contributions of the left and right hemispheres in the evaluation of actions of several second durations. In addition, the differences in temporal judgements of these actions with respect to non-biological movements were investigated, and the role of forced embodiment of the upper limb seen was specifically manipulated (see Fig. SM5 for a summary).

Previous studies have documented differences in supra-seconds and sub-seconds time processing in terms of cognitive mechanisms involved^75,76^. Indeed, while the processing of sub-seconds durations seems to rely on sensory and perceptual mechanisms, other higher-cognitive networks are recruited in the perception of longer, supra-seconds intervals (^77^ for a review). As we were interested in the contribution of higher cognitive functions linked to actions, and also due to the typology of stimuli (whole transitive actions) this study focused on supra-seconds temporal estimation.

Taken as a whole, the results suggest that, at least with reference to these supra-second durations, both the two hemispheres contribute, but with significant differences related with the presence of actions and the embodiment of the body part involved in the movement. The results will be discussed in detail in the following sections.

### Left and right hemispheres in temporal estimation of actions

The role of the two hemispheres in temporal estimations has been identified by means of the comparison of the three patient groups’ performances with respect to the control group. This allowed us to take into account a range of errors that also healthy people make in this typology of tasks. Furthermore, a preliminary analysis shows the same patterns of responses for the three different durations in all the groups, thus excluding the possibility that patients did not identify differences among the three durations.

In the temporal estimation of actions (the ATE task) LBD patients fail when compared to controls, as they underestimate durations, in particular when the actions last more than 4000 ms (4500 ms and 6500 ms). In contrast, right brain-damaged patients (with or without anosognosia for hemiplegia) perform like the healthy control group when judging action durations. Completely different is the pattern of results in the MTE, when the request is the time estimation of a non-biological movement, with no references to human actions. In this case, only patients with lesions in the right hemisphere (RBD and AHP) significantly overestimate the timing of the short-lasting (3000 ms) movement.

A double complementarity seems to emerge from these data. First, the two hemispheres respond in a different way for shorter (3000 ms) and longer (4500–6500) durations, with a role that might be prevalent for the right hemisphere in the former and for the left hemisphere for the latter. Second, these differences do not refer exclusively to the duration but also to the presence or absence of actions. Indeed, while LBD fail in the ATE task (when an action is shown), the RBD fail in the MTE control task (when there is a moving geometrical shape). These results are consistent with the role of the left hemisphere in actions and suggest the possibility that the left hemisphere plays a role in the perception of action sequence timing. To support this hypothesis there is the result of common activations in the left premotor cortex for motor planning and processing of specific durations ordered in time^78^. Nevertheless, it is worth to note that in our patients we do not find any correlations between the number of errors in the apraxia test and the performance in the ATE task. Further studies, with more in-depth assessment of the various forms of apraxia (i.e. ideative and ideomotor apraxia) are needed in order to better understand any potential role of action planning in estimation of temporal aspects of movements and actions.

It is known that the left hemisphere is involved in rhythm perception^41,42,78–80^ and the participation of Broca’s area in rhythm monitoring is considered as an index of the specialization of this hemisphere for temporal integration of rapid stimuli^76^. Thus, and following the *beat frequency model of timing*^24,39–41^, a possibility is that the errors in LBD patients are due to a difficulty in synchronizing the internal representation of actions with the actions seen, rather than to a disorder in gesture planning (i.e. ideomotor apraxia).

In the absence of action, and when an object is moving (MTE), are the right brain-damaged patients who make errors in temporal estimation, with overestimation of the shorter durations. While our explorative lesional analysis confirms a role of right fronto-parietal and insular networks in temporal estimation^9,23–29^, no effects of anosognosia for hemiplegia was found in this task, with AHP who do not differ in respect to RBD. However, we cannot exclude that the unaware patients recruited in this study might have had some degrees of implicit awareness^81–83^ that could emerge only via a more in-depth awareness investigation^84^. Previous studies^81–84^ have demonstrated that there are patients who, although verbally denying their motor impairments (e.g. declaring to be able to move their arms or to walk), behave as if they are aware about their paralysis at some levels (i.e. they spontaneously adopt motor strategies to compensate for their hemiplegia in everyday tasks^85^).

Results in MTE confirm previous lesional studies^49,85–89^ demonstrating a role of the right hemisphere in the processing of supra-seconds durations, probably due to its involvement in working memory and attentional functions. Following the *internal clock* model^33–35^ this reduction of attention (in our group confirmed by MMSE and neglect tests) should impact on the judgement of temporal duration. Another possibility is that in the 3000 ms duration, when the action is perceived faster than in 4500 and 6500 ms, the perceived time results to be expanded. Indeed, the presence of movement has been shown to impact temporal perception, just inducing a temporal dilatation in the perception of faster than slower speeds^90–92^.

Crucially for the aim of this study is that the presence of an action has opposite effects in the left and right brain damaged patients. Indeed, while the presence of actions causes LBD patients to fail the task (ATE), the right hemisphere lesioned patients (RBD and AHP) make larger estimation errors than controls in the MTE task but not in the ATE task. To better understand this result, the effects of embodiment processes were specifically investigated.

### The effects of embodiment in temporal perception of actions

The results from the first experiment, indicating a role of action in temporal estimation, open to the question regarding the possibility that different responses in ATE and MTE tasks are at least in part due to other cognitive processes, in particular to a mechanism of embodiment of the body part (i.e. the upper limb) seen in the video^93^. For this, a second experiment was carried out, where the embodiment processes were manipulated in the direction of reduction, through the removal of the body part that executed the action (ATE-No hand), or in the sense of empowerment, through specific instructions that led the person to imagine that the body part seen was his own (ATE-Embodiment).

The results (see Fig. SM5 and SM7 for a summary of the findings) indicate that in general, the embodiment induced by means of verbal instructions helps the judgments of actions durations, as the three groups do not show significant differences with respect to control in the ATE- Embodiment task. However, when analysing the single groups in the within subject comparisons, data show that embodiment modulates temporal estimation of action only in right damaged patients (RBD and AHP), while this does not have any effects on the LBD performance. More specifically, when the action is seen without the effector (i.e. the upper limb) and thus embodiment is not present, RBD and AHP respond in a similar way as in the MTE task, as if the movement seen is not an human action but rather a non-biological movement. In addition, in a subset of patients, the forced embodiment shows to be effective in right brain damaged patients, who only in this task underestimate temporal duration (in respect to their responses in ATE). Considering the performance of this group in the different tasks, a facilitatory role of the body in temporal estimation emerges, as they: (i) slow down (overestimation) non-biological movements (MTE) and actions in the absence of the body (ATE-No hand); (ii) become more precise when there is the body part performing the action (ATE), and (iii) even accelerate (underestimation) the duration when they imagine that the body part is their own (ATE-embodiment). In contrast, for the LBD performances are the same as in the ATE task, with no differences due to the presence or absence of the body part. Further investigations are needed to understand if this underestimation in the embodiment forced condition may be in some way associated with motor constraints^94^, as in our study we do not find any differences associated with the laterality (correspondence with healthy or motor impaired upper limbs) of the hands seen in the video.

The result of the embodiment effect in right brain damaged patients is in line with previous studies that identify right brain structures as the main network for embodiment mechanisms, and with clinical reports of abnormal embodiment manifestations^95,96^. The pathological embodiment of external body parts has been described^97–99^ in right hemisphere damaged patients, when they observe and external arm (i.e. the examiner’s upper limb) that is placed in a congruent position with respect to the patient’s homologue body part^100^, also in the absence of specific body representations disorders (e.g. asomatognosia or somatoparaphrenia^101^). The symptom is associated with white matter lesions involving the corona radiata and the superior longitudinal fasciculus^99^. This sensitivity to embodiment manipulations may be used in these patients with rehabilitative aims, in order to improve awareness of deficits^65,66,102^ or motor control on upper limbs^84,85^. Future studies may further explore the effect of embodiment mechanism on time estimations of actions via verbal embodiment questionnaires.

Finally, the lack of impact of bodily-related stimuli (the presence or absence of the body part) and embodiment in LBD responses suggest that these patients base their responses on the direct perception or representation of the action, rather on body representations.

### Limitations and conclusions

A first limitation of the study is the limited number of participants and the different numbers in the various groups. This was due to technical reasons, as the tasks were time-consuming and multiple sessions were needed. Some patients did not carry out both the experiments mainly due to clinical reasons (e.g. patient’s covid infection, general health problems, rescheduling of rehabilitation activities or patients’ discharge). Although this suggests caution in the interpretation of results, the small number of patients is usually accepted in neuropsychological studies, in particular when using challenging experimental paradigms that require a good level of cognitive functioning and tolerance to long tasks. However, in the four tasks that we administered, the groups’ performances were consistent, and this certainly represents a guarantee with respect to the solidity of the data. This consistency in the pattern of results allows us to consider that the inevitable differences between the stimuli in the different conditions (e.g. the presence of the hand that is missing in No-Hand condition, the non-biological stimulus in MTE that is indeed really different in respect to the biological motion conditions) do not have a significant impact in explaining the differences between the participants’ groups. Secondly, for some of the patients, neuroimaging data were not available and this prevented us from analyzing the neural correlates of patients with left lesions and allowed only an exploratory analysis for right-damaged patients.

Finally, the neuropsychological assessment used as a preliminary screening of apraxia might have limited the investigation of the potential role of this syndrome in the estimation of action durations. Indeed, this test excluded severe apraxia symptoms in our sample and did not allow for the investigation of a subgroup of LBD apraxic patients. Future studies involving patients suffering from apraxia will help to understand the role of action planning in action time estimation.

In summary, the study indicates a joint, complementary contribution of left and right hemispheres in temporal estimations of supra-second actions, referred both to different durations (with left hemisphere involved more in 4500 and 6500 ms actions and right one in 3000 ms) and to the presence of actions or no-biological movements. While LBD base their judgements on action or movement per se, RBD estimate time of actions basing on body representations. Cognitive functions associated with motricity, in particular anosognosia for hemiplegia and apraxia, do not seem to have specific effects on temporal estimation.

## Material and methods

Two experiments were carried out in the study, that were aimed at investigating the patients’ abilities to estimate various durations of actions and the potential role of embodiment in temporal estimation, respectively. All the participants signed the informed consent, and the research was conducted in accordance with the guidelines of the Declaration of Helsinki (2013) and approved by the Local Ethical Committee (CESC – Comitato Etico Studi Clinici delle Provincie di Verona e Rovigo, Prot. 2019-08).

### Experiment 1: Action time estimation task (ATE)

The abilities of action timing estimation were assessed by comparing the performance of a sample of stroke patients (N = 33) with those of a healthy control group. Participants performed an experimental task, the Action Time Estimation (ATE) task and a control task, namely the Movement Time Estimation (MTE), where a non-biological movement was shown. The two tasks were administered in a balanced order.

### Participants

Clinical and experimental data from 33 (all hospitalized) patients suffering from unilateral brain damage (n.20 right hemisphere- RBD and n. 13 left hemisphere- LBD) were collected. 42 healthy controls served as control and were matched for age (t = 1.47; df = 74; *p* = 0.15) and education (t = 0.24; df = 74; *p* = 0.81). The inclusion criteria were the presence of: (i) unilateral hemisphere damage, secondary to a first-ever stroke, as confirmed by clinical neuroimaging; (ii) motor deficit of contralesional upper limb (scores ≤ 50 for both the upper and lower limbs at the Motricity Index scale^103,104^); (iii) right-handedness (Edinburgh Handedness Inventory, EHI^105^). Exclusion criteria were the presence of: (i) bilateral brain damage; (ii) severe comprehension deficits (Token test and Oral and Written Comprehension, AAT^68^); (iii) severe general cognitive impairment (MMSE^67^; italian validations^106,107^); (iv) mood disturbance that precluded completion of the study.

### Preliminary neuropsychological assessment

Table 1 reports clinical and neuropsychological data. As the assessment for Anosognosia for Hemiplegia (^70^ see below for the description) identified the presence of unawareness for motor impairment in 6 patients, the RBD group was divided into two groups of patients with (AHP) and without (RBD) anosognosia for hemiplegia. In this way, there were 4 groups: LBD (n.13) AHP (n. 7), RBD (n.13), C (n. 42). In the patients, the presence of apraxia was explored via the Ideomotor apraxia test (Feinberg, 2000) and short-term verbal memory impairment by means of the digit span score (forward^72^; Italian validation^108^). Personal neglect was assessed by means of the ‘Comb’ subtest of the ‘Comb and Razor/Compact test’^74^, while the scores in the Line Crossing and Line Bisection subtests of the BIT (Behavioral Inattention Test^73^) were used as a measure of extra-personal neglect. Hemianopsia was excluded by means of clinical examination.

To explore any differences in the neuropsychological profile among the three groups of patients (AHP, RBD and LBD), linear models were computed for each test.

### Assessment of anosognosia for hemiplegia

The presence of AHP was assessed by means of two tests, assessing respectively the patients’ acknowledge of their ability in in daily life activities (VATA-m test^70^) and their judgement about their current ability to move the upper limb^71^.

In the VATA-m test^70^ patients are requested to look at some images showing unilateral and bilateral upper or lower limb actions of daily life and to rate their current ability to perform these actions (from 0 = no problem to 3 = severe problem). Only the VATA-m for the upper limb was here considered. This is based on 8 trials, with scores ranging from 0 to 24. Patient’s responses are then compared to the caregiver or physiotherapist’s judgments and the patients-examiner discrepancy is considered for the diagnosis (for the upper limb, range: − 24/ + 24, scores 3.3–8 = mild anosognosia; 8.1–16 = moderate anosognosia, and > 16.1 = severe anosognosia).

In the Bisiach’s scale^71^ patients are interviewed about their current ability to move their upper limb and the eventual reasons for their deficits. In the version of the test here used, the following partially modified scoring of the scale was adopted^102^. As in the original version, a “0” score indicated spared consciousness of the disease (= the disorder is spontaneously reported or mentioned by the patient following a general question about his/her complaints) and a “1” score was assigned when patients referred to their disability only after specific questions about the strength of their left limbs. A “1,5” score (not reported in the original version of the scale, but previously used in studies on AHP; e.g.^84,102^) indicated that general deficits and some motor impairments were reported, but these were not attributed to the presence of hemiplegia (e.g., a previous unrelated surgical operation or arthrosis). Finally, patients scoring ‘2’ or ‘3’ were considered to be anosognosic, as their awareness of the disease emerged only after a demonstration through a routine technique of neurological examination (i.e., “please raise your left arm”, score 2) or not emerge at all (score 3).

This double assessment of AHP allowed us to take into account the potential variability of AHP symptoms in time and in relation to the context of the questioning^62,109^.

To explore potential differences in the neuropsychological profile among the three groups, linear regression models have been computed for each test. The results of the preliminary neuropsychological assessment referring to the samples of Experiment 1 are described in SM1.

### Action time estimation task (ATE): stimuli and procedure

In the ATE condition (Fig. 1a) some videos were shown, where a left or right hand were presented while using an object (in order to avoid any difference between the two hands the same video recorded for the right hand was mirrored for left hand condition). Actions were presented in a first-person perspective and followed a vertical trajectory (from bottom to top). These were: closing a fan; cleaning a stain with a sponge; wrapping up a tape; tearing off a velcro. Each of these actions was recorded (Canon EOS600D) once and the videos were then edited on Windows Movie Maker in order to manipulate the action velocity and present them in one of three different durations: 3000 ms, 4500 ms, 6500 ms (for both the left and right hand, with the coincidence of video and movement durations). The video's frame size was 1080 × 720 pixels, the encoded frame per second were kept constant (efps: 60) and the stimuli were black and white coloured, to prevent colour-driven attentional orienting^110^ (Fig. 1a and examples at https://osf.io/u7wjc/).

Furthermore, each video was presented three times, with a total of 72 trials (4 × 3 × 3 × 2). These were divided into four blocks (18 trials in each). Actions were presented in a semi-randomized order (E-prime software) to avoid two consecutive displays of the same object and the same duration.

Instructions were verbally given while the same written instructions were appearing at the centre of the screen. Participants were required to carefully look at the stimuli in the centre of the screen and to estimate the duration of the action at the end of each trial, when a question was asked (‘How long did the action last?’). Responses were given indicating the action duration on a graduated scale (range 0–8 s, with intervals indicating 0.5 s; SM6). Patients were explicitly asked not to count seconds during the observation of the action. The three durations were unevenly separated of 1.5 s (between 3000 and 4500 ms durations), 2 s (between 4500 and 6500 ms durations), and 3.5 s (between 3000 and 6500 ms durations) to additionally prevent counting. After each patient’s response, a new video appeared on the screen. A short preliminary training (four trials) was administered to practice and three breaks were programmed to avoid attentional fatigue.

### Movement time estimation task (MTE): stimuli and procedure

1. The Movement Time Estimation task (Fig. 2a) was implemented to control for the patients’ general abilities to estimate time durations in the context of a target-directed non-biological movement (i.e. without actions). The MTE task was analogous to the ATE task, in procedure, durations of the movement seen, and modality of response (the graduate scale) but different in stimuli. These consisted of videos showing a black circle moving along a vertical trajectory (from bottom to up) toward a bar. Similarly, as in the experimental task, the video durations were manipulated in a way that the circle movement might last 3000 ms, 4500 ms or 6500 ms (efps: 60). At the end of each video, participants were required to estimate the duration of the movement. Each duration was presented 6 times with a total of 18 trials. Three practice trials were administered before the beginning of the task.

### Statistical analyses

The Shapiro–Wilk normality test showed that the normality assumption is respected for both the error-indexes of ATE (W = 0.997, *p*-value = 0.096) and MTE (W = 0.995, *p*-value = 0.68).

Statistical analyses were carried out in three steps^111^. At first, the ability in discriminating the three different durations was ascertained in each group. Then, the three groups of patients were compared with controls in the estimation of action durations. Finally, correlations of results with clinical variables were analysed.

To quantify the errors in the estimation of durations in both ATE and MTE tasks, an index of “estimation error” was computed for each response, as the difference between the participant’s subjective estimation and the actual duration of action/movement. In this way, an error near zero indicates an accurate estimation, an error above zero shows overestimation, and an error below zero indicates an underestimation of the duration.

Missing responses were excluded from the analysis (ATE = 0.01%; MTE = 0.8%) as well as the outlier responses, which were identified on a subjective basis (i.e. ± 2 standard deviations from the mean of each subject, in each condition for each duration). For technical problems, one AHP patient did not perform the MTE task.

The participants’ ability to identify the three durations of the movement was explored by means of a linear mixed-effects model (lme4 package^112^) considering the responses given by the four groups in the ATE experimental task and in the MTE control task as dependent variables, with Group (C, RBD, LBD, AHP) and Duration (3000, 4500, 6500) as fixed effects and including the Duration variable and the subjects as random effects. Post-hoc analyses were computed on the model (emmeans package^113^), using Tukey method for multiple comparison adjustments. Differences among the groups emerged with respect to the Duration (see SM2), and thus the comparisons between patients’ groups and controls were carried out separately for each Duration.

Furthermore, a preliminary analysis for the ATE task investigated the differences in estimation errors between videos showing left- and right-hand actions, within and between each group by means of a linear mixed-effects model (lme4 package^112^). The estimation errors given by the four groups in the ATE task were included as dependent variables, with Group (C, RBD, LBD, AHP) and Effector (right hand, left hand) as fixed effects and including the Effector variable and the subjects as random effects. As no effect of the Effector emerged (F = 0.95, *p* = 0.33), nor the interaction between group and Effector (F = 0.75, *p* = 0.53), the errors regarding the left- and right-hand videos were averaged for each subject, for each Duration.

For the second step of the analysis, the errors in estimation (i.e. the difference between the duration estimated and the actual duration) were analysed via pairwise *t*-tests (pairwise.*t*.test function), confronting the responses of each group of patients with healthy controls.

Finally, Pearson's correlations have been computed between the errors for each task, for each duration, and the clinical data where a difference between the groups was found, via the corr.test function (see SM4 for the correlations results).

### Experiment 2: ATE-embodiment

The second experiment aimed at exploring the potential effect of the embodiment of the hand seen in the videos in modulating temporal estimation of actions. This was investigated by means of two complementary tasks (ATE-No hand and ATE-embodiment), where embodiment was manipulated.

### Participants

While the whole group of subjects participated in the ATE-No hand task, the ATE-Embodiment task was administered only to a subgroup of participants. In fact, in order to avoid delayed effects of manipulation on the subsequent task, the ATE-Embodiment was administered after the ATE-No hand task. This means that some patients (and controls) did not complete the study for various different reasons (e.g. discharge from the hospital, limited time available, fatigue).

The final group for the ATE-Embodiment task was made up of 16 patients (n. 5 LBD n.7 RBD, n. 4 AHP) and 16 healthy controls, who were matched for age (t =  − 1.27; df = 30; *p* = 0.2) and education (t = 1.21; df = 30; *p* = 0.24; the demographic and clinical data of the control and patients groups are shown in SM5).

### Action time estimation-no hand (ATE-no hand) and action time estimation-embodiment (ATE-embodiment) tasks: stimuli and procedure

In the ATE-no hand task, the same stimuli as in the ATE were shown with the only difference that the objects were moved without the presence of an effector and the actions were seen as if the objects were self-animated (Fig. 4a and example at https://osf.io/u7wjc/). Three blocks of 16 trials were presented in this condition, with each stimulus presented 4 times for each duration, in a semi-randomized order. In the ATE-Embodiment task, the task and the number of stimuli were the same as the ATE in all but the instruction (Fig. 5a), that explicitly asked to imagine as if the hand seen in the video was their own hand (i.e. ‘please, imagine the hand performing the action as your own’).

### Statistical analysis

As for the previous experiment, the error-index was computed for each participant’s response and missing (0.01%) and outlier responses were excluded from the analyses. The Shapiro–Wilk normality test showed that the normality assumption is respected for both the error-indexes of ATE-embodiment (W = 0.99, *p*-value = 0.93) and ATE-NH (W = 0.99, *p*-value = 0.58).

Participants’ ability to identify the three durations was investigated as described in the statistical analysis of the Experiment 1, by means of two linear mixed-effects models (for the ATE-NH and ATE-Embodiment tasks separately) considering the responses given by the four groups with Group (H, RBD, LBD, AHP) and Duration (3000, 4500, 6500) as fixed effects and including the Duration variable and the Subjects as random effects. The overall analysis of the model was computed via the Kenward–Roger test and post-hoc analyses were computed on the model (emmeans package^113^), using Tukey method for multiple comparison adjustments.

Preliminary comparisons have been conducted within each group between the errors of the left- and right- hand actions of the embodiment task. As in the ATE task, also in the embodiment version no difference between the two hands was found, and thus the errors of the left- and right-hand actions were averaged for each subject and duration.

In order to explore the potential effects of the embodiment induced by the new instructions, the data of the ATE task (of patients who also participated in the Experiment 2) and the ATE-Embodiment task were analysed via linear mixed-effects models (lme4 package^111^) which allowed us to investigate within-group and between groups (controls versus each patient group) differences between the ATE task and ATE-No Hand and ATE-Embodiment tasks separately. Given our interest in the investigation of between-group differences specific for controls versus each patient group, the models were computed separately for each control versus patient group contrast (Group variable), for each duration. Thus, the models included the errors in the (i) ATE and ATE-No Hand, and (ii) ATE and ATE-Embodiment tasks as dependent variables, with Group and Condition (ATE, ATE-No Hand; ATE, ATE-Embodiment) as fixed effect and including Condition and subjects as random effect. Post-hoc analyses were computed on the model (emmeans package^113^), using Tukey method for multiple comparison adjustments.

### Lesional analyses

The brain lesions images of 9 RBD/AHP patients were acquired via computerised tomography (CT) or magnetic resonance imaging (MRI) and analized following a manual procedure^62^. Lesions from these scans were segmented and co-registered using a manual procedure. Lesions were outlined by two of the experiments who were blind to each scan's group classification and patients scores in the experimental tasks. In the case of disagreement of two lesion plots, the opinion of a third, expert anatomist was requested. Scans were registered to the T1-weighted MRI scan template (ICBM152) of the Montreal Neurological Institute, furnished with the MRIcron software (ch2, http://www.cabiatl.com/mricro/mricron/index.html). First, the standard template (size: 181 217 181 mm, voxel resolution: 1 mm^2^) was rotated on the three planes in order to match the orientation of the patient's MRI or CT scan. Lesions were outlined on the axial slices of the rotated template. The resulting lesion volumes were then rotated back into the canonical orientation, in order to align the lesion volumes of each patient to the same stereotaxic space. Finally, in order to exclude voxels of lesions outside white and gray matter brain tissue, lesion volumes were filtered by means of custom masks based on the ICBM152 template. To predict lesion location associated with the deficit in short duration estimation of non-biological movement, the manually drawn lesions were used as dependent variables in a regression analysis, and the errors in the 3000 ms duration of the MTE task were used as independent variable. Threshold-Free Clusters Enhancement option was applied to boost cluster-like structures of voxels and results that survived 1000 permutations testing were controlled for family-wise error rate (*p* < 0.05).

## Supplementary Information

Supplementary Information.

## Data Availability

The dataset analysed during the current study is available at https://osf.io/u7wjc/.

## References

[1] Hancock PA (2020). Time–our greatest tool: do we design with respect to time, or is it that we can design time itself?. Ergon. Des..

[2] Oestreicher C (2022). The manifold definitions of time. Dialogues Clin. Neurosci..

[3] Zhang D, Liu Y, Wang X, Chen Y, Luo Y (2014). The duration of disgusted and fearful faces is judged longer and shorter than that of neutral faces: The attention-related time distortions as revealed by behavioral and electrophysiological measurements. Front. Behav. Neurosci..

[4] Droit-Volet S, Meck WH (2007). How emotions colour our perception of time. Trends Cogn. Sci..

[5] Cáceda R (2020). Slower perception of time in depressed and suicidal patients. Eur. Neuropsychopharmacol..

[6] Donev IS, Ivanova MS, Conev NV (2020). Fast time perception is associated with high levels of anxiety in cancer patients prior to starting chemotherapy. Biosci. Trends.

[7] Biermann T (2011). Time perception in patients with major depressive disorder during vagus nerve stimulation. Pharmacopsychiatry.

[8] Crisci C, Caccavale M, Trojano L (2016). Aging and the subjective experience of time. Aging Clin. Exp. Res..

[9] Trojano L, Caccavale M, De Bellis F, Crisci C (2017). The brain and the subjective experience of time. A voxel based symptom-lesion mapping study. Behav. Brain Res..

[10] Teghil A, Di Vita A, Pietranelli V, Matano A, Boccia M (2020). Duration reproduction in regular and irregular contexts after unilateral brain damage: Evidence from voxel-based lesion-symptom mapping and atlas-based hodological analysis. Neuropsychologia.

[11] Moore JW, Obhi SS (2012). Intentional binding and the sense of agency: a review. Conscious. Cogn..

[12] Nather FC, Bueno JLO (2011). Static images with different induced intensities of human body movements affect subjective time. Percept. Mot. Skills.

[13] Botvinick M, Cohen J (1998). Rubber hands ‘feel’ touch that eyes see. Nature.

[14] Blanke O, Metzinger T (2009). Full-body illusions and minimal phenomenal selfhood. Trends Cogn. Sci..

[15] Scandola M (2014). Rubber hand illusion induced by touching the face ipsilaterally to a deprived hand: evidence for plastic “somatotopic” remapping in tetraplegics. Front. Hum. Neurosci..

[16] De Kock R, Gladhill KA, Ali MN, Joiner WM, Wiener M (2021). How movements shape the perception of time. Trends Cogn. Sci..

[17] Binetti N (2015). Binding space and time through action. Proc. R. Soc. B Biol. Sci..

[18] Tomassini A, Morrone MC (2016). Perceived visual time depends on motor preparation and direction of hand movements. Sci. Rep..

[19] Teki S (2016). A citation-based analysis and review of significant papers on timing and time perception. Front. Neurosci..

[20] Hayashi MJ, Kantele M, Walsh V, Carlson S, Kanai R (2014). Dissociable neuroanatomical correlates of subsecond and suprasecond time perception. J. Cogn. Neurosci..

[21] Perbal-Hatif S (2022). A neuropsychological approach to time estimation. Dialogues Clin. Neurosci..

[22] Ivry RB, Spencer RMC (2004). The neural representation of time. Curr. Opin. Neurobiol..

[23] Macar F (2002). Activation of the supplementary motor area and of attentional networks during temporal processing. Exp. Brain Res..

[24] Coull JT, Vidal F, Nazarian B, Macar F (2004). Functional anatomy of the attentional modulation of time estimation. Science.

[25] Mayo JP, Sommer MA (2013). Neuronal correlates of visual time perception at brief timescales. Proc. Natl. Acad. Sci. U. S. A..

[26] Cope TE, Grube M, Singh B, Burn DJ, Griffiths TD (2014). The basal ganglia in perceptual timing: timing performance in Multiple System Atrophy and Huntington’s disease. Neuropsychologia.

[27] Teghil A (2019). Neural substrates of internally-based and externally-cued timing: an activation likelihood estimation (ALE) meta-analysis of fMRI studies. Neurosci. Biobehav. Rev..

[28] Monfort V (2014). Distortion of time interval reproduction in an epileptic patient with a focal lesion in the right anterior insular/inferior frontal cortices. Neuropsychologia.

[29] Wittmann M, Simmons AN, Aron JL, Paulus MP (2010). Accumulation of neural activity in the posterior insula encodes the passage of time. Neuropsychologia.

[30] Palombo DJ (2020). The human medial temporal lobe is necessary for remembering durations within a sequence of events but not durations of individual events. J. Cogn. Neurosci..

[31] Lee ACH, Thavabalasingam S, Alushaj D, Çavdaroğlu B, Ito R (2020). The hippocampus contributes to temporal duration memory in the context of event sequences: a cross-species perspective. Neuropsychologia.

[32] Melgire M (2005). Auditory/visual duration bisection in patients with left or right medial-temporal lobe resection. Brain Cogn..

[33] Treisman M (1963). Temporal discrimination and the indifference interval. Implications for a model of the ‘internal clock’. Psychol. Monogr..

[34] Church RM (1984). Properties of the internal clock. Ann. N. Y. Acad. Sci..

[35] Macar, F. Time psychophysics and related models, in *Time, mind, and behavior* 112–130. (1985).

[36] Allman MJ, Teki S, Griffiths TD, Meck WH (2014). Properties of the internal clock: first- and second-order principles of subjective time. Ann. Rev. Psychol..

[37] Wiener M, Turkeltaub P, Coslett HB (2010). The image of time: a voxel-wise meta-analysis. Neuroimage.

[38] Marinho V (2019). Impaired decision-making and time perception in individuals with stroke: behavioral and neural correlates. Rev. Neurol..

[39] Matell MS, Meck WH (2004). Cortico-striatal circuits and interval timing: coincidence detection of oscillatory processes. Brain Res. Cogn. Brain Res..

[40] Matell MS, Meck WH (2000). Neuropsychological mechanisms of interval timing behavior. Bioessays.

[41] Buhusi CV, Meck WH (2005). What makes us tick? Functional and neural mechanisms of interval timing. Nat. Rev. Neurosci..

[42] Coull JT, Cheng RK, Meck WH (2010). Neuroanatomical and neurochemical substrates of timing. Neuropsychopharmacology.

[43] Stevens MC, Kiehl KA, Pearlson G, Calhoun VD (2007). Functional neural circuits for mental timekeeping. Hum. Brain Mapp..

[44] Merchant H, Harrington DL, Meck WH (2013). Neural basis of the perception and estimation of time. Annu. Rev. Neurosci..

[45] Merchant H, Zarco W, Prado L (2008). Do we have a common mechanism for measuring time in the hundreds of millisecond range? Evidence from multiple-interval timing tasks. J. Neurophysiol..

[46] Fontes R (2016). Time perception mechanisms at central nervous system. Neurol. Int..

[47] Low E (2016). Beyond neglect: preliminary evidence of retrospective time estimation abnormalities in non-neglect stroke and transient ischemic attack patients. Sci. Rep..

[48] Basso G, Nichelli P, Frassinetti F, Di Pellegrino G (1996). Time perception in a neglected space. NeuroReport.

[49] Danckert J (2007). Neglected time: impaired temporal perception of multisecond intervals in unilateral neglect. J. Cogn. Neurosci..

[50] Calabria M (2011). Time perception in spatial neglect: a distorted representation?. Neuropsychology.

[51] de Montalembert M, Mamassian P (2012). Processing temporal events simultaneously in healthy human adults and in hemi-neglect patients. Neuropsychologia.

[52] Harrington DL, Haaland KY, Knight RT (1998). Cortical networks underlying mechanisms of time perception. J. Neurosci..

[53] Merrifield C, Hurwitz M, Danckert J (2010). Multimodal temporal perception deficits in a patient with left spatial neglect. Cogn. Neurosci..

[54] Kurosaki Y, Terasawa Y, Ibata Y, Hashimoto R, Umeda S (2020). Retrospective time estimation following damage to the prefrontal cortex. J. Neuropsychol..

[55] Cubelli R (2017). Definition: apraxia. Cortex.

[56] Canzano L, Scandola M, Pernigo S, Aglioti SM, Moro V (2014). Anosognosia for apraxia: experimental evidence for defective awareness of one’s own bucco-facial gestures. Cortex.

[57] Scandola M (2021). Gesture errors in left and right hemisphere damaged patients: a behavioural and anatomical study. Neuropsychologia.

[58] Scandola M (2021). Anosognosia for limb and bucco-facial apraxia as inferred from the recognition of gestural errors. J. Neuropsychol..

[59] Babinski, J. F. F. Contribution à l’Étude des Troubles Mentaux dans l’Hémiplégie Organique Cérébrale (Anosognosie). *Rev. Neurol.* (1914) 10.1016/j.cortex.2014.04.019 25481462

[60] Karnath H-O, Baier B, Nägele T (2005). Awareness of the functioning of one’s own limbs mediated by the insular cortex?. J. Neurosci..

[61] Pacella V (2019). Anosognosia for hemiplegia as a tripartite disconnection syndrome. Elife.

[62] Moro V (2016). Motor versus body awareness: voxel-based lesion analysis in anosognosia for hemiplegia and somatoparaphrenia following right hemisphere stroke. Cortex.

[63] Berti A (2005). Shared cortical anatomy for motor awareness and shared cortical anatomy for motor awareness and motor control. Science.

[64] Romano D (2021). Back in control of intentional action: Improvement of ideomotor apraxia by mirror box treatment. Neuropsychologia.

[65] Besharati S, Kopelman M, Avesani R, Moro V, Fotopoulou A (2015). Another perspective on anosognosia: self-observation in video replay improves motor awareness. Neuropsychol. Rehabil..

[66] Fotopoulou A, Rudd A, Holmes P, Kopelman M (2009). Self-observation reinstates motor awareness in anosognosia for hemiplegia. Neuropsychologia.

[67] Folstein MF, Folstein SE, McHugh PR (1975). “Mini-mental state”: a practical method for grading the cognitive state of patients for the clinician. J. Psychiatr. Res..

[68] Luzzatti, C. *et al.* New normative data for the Italian version of the Aachen Aphasia Test (A.A.T). (1994).

[69] Feinberg TE, Roane DM, Ali J (2000). Illusory limb movements in anosognosia for hemiplegia. J. Neurol. Neurosurg. Psychiatry.

[70] Della Sala S, Cocchini G, Beschin N, Cameron A (2009). VATA-m: visual-analogue test assessing anosognosia for motor impairment. Clin. Neuropsychol..

[71] Bisiach E, Vallar G, Perani D, Papagno C, Berti A (1986). Unawareness of disease following lesions of the right hemisphere: anosognosia for hemiplegia and anosognosia for hemianopia. Neuropsychologia.

[72] Baddeley AD, Thomson N, Buchanan M (1975). Word length and the structure of short-term memory. J. Verbal Learn. Verbal Behav..

[73] Wilson B, Cockburn J, Halligan P (1987). Development of a behavioral test of visuospatial neglect. Arch. Phys. Med. Rehabil..

[74] McIntosh RD, Brodie EE, Beschin N, Robertson IH (2000). Improving the clinical diagnosis of personal neglect: a reformulated comb and razor test. Cortex.

[75] Koch G, Oliveri M, Caltagirone C (2009). Neural networks engaged in milliseconds and seconds time processing: evidence from transcranial magnetic stimulation and patients with cortical or subcortical dysfunction. Philos. Trans. R. Soc. Lond. B. Biol. Sci..

[76] Lewis PA, Miall RC (2003). Distinct systems for automatic and cognitively controlled time measurement: evidence from neuroimaging. Curr. Opin. Neurobiol..

[77] Mioni G, Grondin S, Bardi L, Stablum F (2020). Understanding time perception through non-invasive brain stimulation techniques: a review of studies. Behav. Brain Res..

[78] Schubotz RI, Friederici AD, Yves von Cramon D (2000). Time perception and motor timing: a common cortical and subcortical basis revealed by fMRI. Neuroimage.

[79] Tallal P, Miller S, Fitch RH (1993). Neurobiological basis of speech: a case for the preeminence of temporal processing. Ann. N. Y. Acad. Sci..

[80] Platel H (1997). The structural components of music perception: a functional anatomical study. Brain.

[81] Cocchini G, Beschin N, Fotopoulou A, Dell Sala S (2010). Explicit and implicit anosognosia or upper limb motor impairment. Neuropsychologia.

[82] Fotopoulou A, Pernigo S, Maeda R, Rudd A, Kopelman MA (2010). Implicit awareness in anosognosia for hemiplegia: unconscious interference without conscious re-representation. Brain.

[83] Moro V, Pernigo S, Zapparoli P, Cordioli Z, Aglioti SM (2011). Phenomenology and neural correlates of implicit and emergent motor awareness in patients with anosognosia for hemiplegia. Behav. Brain Res..

[84] Moro V, Pacella V, Luxon D, Cocchini G (2021). Rehabilitation and modulation aimed at ameliorating awareness in anosognosia for hemiplegia. Acta Neuropsychol..

[85] Moro V, Scandola M, Bulgarelli C, Avesani R, Fotopoulou A (2015). Error-based training and emergent awareness in anosognosia for hemiplegia. Neuropsychol. Rehabil..

[86] Rubia K, Schuri U, Cramon DYV, Poeppel E (1997). Time estimation as a neuronal network property: a lesion study. NeuroReport.

[87] Koch G, Oliveri M, Carlesimo GA, Caltagirone C (2002). Selective deficit of time perception in a patient with right prefrontal cortex lesion. Neurology.

[88] Kagerer FA, Wittmann M, Szelag E, Steinbüchel NV (2002). Cortical involvement in temporal reproduction: evidence for differential roles of the hemispheres. Neuropsychologia.

[89] Piras F (2013). Time dysperception perspective for acquired brain injury. Front. Neurol..

[90] Brown SW (1995). Time, change, and motion: the effects of stimulus movement on temporal perception. Percept. Psychophys..

[91] Tomassini A, Gori M, Burr D, Sandini G, Morrone MC (2011). Perceived duration of visual and tactile stimuli depends on perceived speed. Front. Integr. Neurosci..

[92] Yokosaka T, Kuroki S, Nishida S, Watanabe J (2015). Apparent time interval of visual stimuli is compressed during fast hand movement. PLoS ONE.

[93] Chen YH, Pizzolato F, Cesari P (2013). Observing expertise-related actions leads to perfect time flow estimations. PLoS ONE.

[94] De Kock R, Zhou W, Joiner WM, Wiener M (2021). Slowing the body slows down time perception. Elife.

[95] Aglioti S, Smania N, Manfredi M, Berlucchi G (1996). Disownership of left hand and objects related to it in a patient with right brain damage. NeuroReport.

[96] Moro V, Zampini M, Aglioti SM (2004). Changes in spatial position of hands modify tactile extinction but not disownership of contralesional hand in two right brain-damaged patients. Neurocase.

[97] Pia L, Garbarini F, Fossataro C, Fornia L, Berti A (2013). Pain and body awareness: evidence from brain-damaged patients with delusional body ownership. Front. Hum. Neurosci..

[98] Garbarini F, Fossataro C, Pia L, Berti A (2020). What pathological embodiment/disembodiment tell US about body representations. Neuropsychologia.

[99] Pia L (2020). The anatomo-clinical picture of the pathological embodiment over someone else’s body part after stroke. Cortex.

[100] Fossataro C, Gindri P, Mezzanato T, Pia L, Garbarini F (2016). Bodily ownership modulation in defensive responses: physiological evidence in brain-damaged patients with pathological embodiment of other’s body parts. Sci. Rep..

[101] Jenkinson PM, Moro V, Fotopoulou A (2018). Definition: asomatognosia. Cortex.

[102] D’Imperio D, Bulgarelli C, Bertagnoli S, Avesani R, Moro V (2017). Modulating anosognosia for hemiplegia: the role of dangerous actions in emergent awareness. Cortex.

[103] Collin C, Wade D (1990). Assessing motor impairment after stroke: a pilot reliability study. J. Neurol. Neurosurg. Psychiatry.

[104] Demeurisse G, Demol O, Robaye E (1980). Motor evaluation in vascular hemiplegia. Eur. Neurol..

[105] Oldfield RC (1971). The assessment and analysis of handedness: the Edinburgh inventory. Neuropsychologia.

[106] Magni E, Binetti G, Bianchetti A, Rozzini R, Trabucchi M (1996). Mini-mental state examination: a normative study in Italian elderly population. Eur. J. Neurol..

[107] Measso G (1993). The mini-mental state examination: normative study of an Italian random sample. Dev. Neuropsychol..

[108] Monaco M, Costa A, Caltagirone C, Carlesimo GA (2013). Forward and backward span for verbal and visuo-spatial data: standardization and normative data from an Italian adult population. Neurol. Sci..

[109] Moro V (2021). The Motor Unawareness Assessment (MUNA): a new tool for the assessment of anosognosia for hemiplegia. J. Clin. Exp. Neuropsychol..

[110] Bekhtereva V, Müller MM (2017). Bringing color to emotion: the influence of color on attentional bias to briefly presented emotional images. Cogn. Affect. Behav. Neurosci..

[111] R Core Team. R: a language and environment for statistical computing (2018).

[112] Bates D, Mächler M, Bolker BM, Walker SC (2015). Fitting linear mixed-effects models using lme4. J. Stat. Softw..

[113] Russel, V. L. *et al.* Package ‘emmeans’ type package title estimated marginal means, aka least-squares means (2018).

