# Supplementary material for: Temporal judgments of actions following unilateral brain damage
Source: Sci Rep. 2022 Dec 15;12:21668. doi: 10.1038/s41598-022-26070-9 (PMC9755153; doi:10.1038/s41598-022-26070-9)
Supplement: Supplementary file 1 — Supplementary Information. [file 41598_2022_26070_MOESM1_ESM.docx]

Supplementary Materials

**TEMPORAL JUDGMENTS OF ACTIONS FOLLOWING UNILATERAL BRAIN DAMAGE**

V. Pacella^1,2^, M. Scandola^3^, M. Bà^3^, N. Smania^4^, M. Beccherle^3^, E. Rossato^5^, D. Volpe^6^, V. Moro^3^*

**SM 1. The neuropsychological assessment. Comparison among groups**

The neuropsychological data have been normalised as z-scores. To compare the neuropsychological assessment scores of the three groups of patients, linear models were computed for each test, with the Group as independent variable. The Anova was then computed on the model and, in case of a significant effect, post-hoc analysis was performed via emmeans. A significant difference was found in the MMSE scores (F = .59; df = 2; p = 0.04) with AHP showing lower scores than RBD groups (t=-2.61; df=28; p=0.03). Furthermore, a significant difference was found for the AAT-oral comprehension scores among the three groups (F = 4; df = 2; p = 0.029), with AHP showing lower scores than LBD group (t=-2.66; df=30; p=0.032). The AHP group’s number of errors in the Token test is significantly higher than the RBD (t=4.07; df=30; p < 0.001) and LBD (t=2.46; df=30; p = 0.05) groups’ one.

Both RBD and AHP performed worse than LBD in the Line Bisection test (t=-2.86; df=30; p=0.02 and t=-3.8; df=30; p=0.002, respectively). Furthermore, the Line crossing test post-hoc revealed a significant difference between the AHP and the LBD groups (t= 7.85; df=30; p<0.001). As expected, the scores in the VATA-m were significantly lower in AHP than RBD g (t=8.26; df=30; p<0.001), and LBD groups (t= 7.85; df=30; p<0.001), confirming the presence of anosognosia for motor abilities in the AHP group.

**SM 2. Identification of the three durations (3000 ms, 4500 ms, 6500 ms)**

All four groups could distinguish among the three durations, as confirmed by the significant interaction between Group and Duration (F = 11.56; df = 6; p < 0.001), The post-hoc analyses (Table SM1) confirm the different estimations given to the three durations by each group (Figure SM1a). A similar result is found in MTE, where the model identifies a significant interaction between Group and Duration (F = 4.19; df = 6; p < 0.001) and the post-hoc analysis (Table SM 2) show a significant difference among the errors of the three durations for each group (Figure SM 1b).


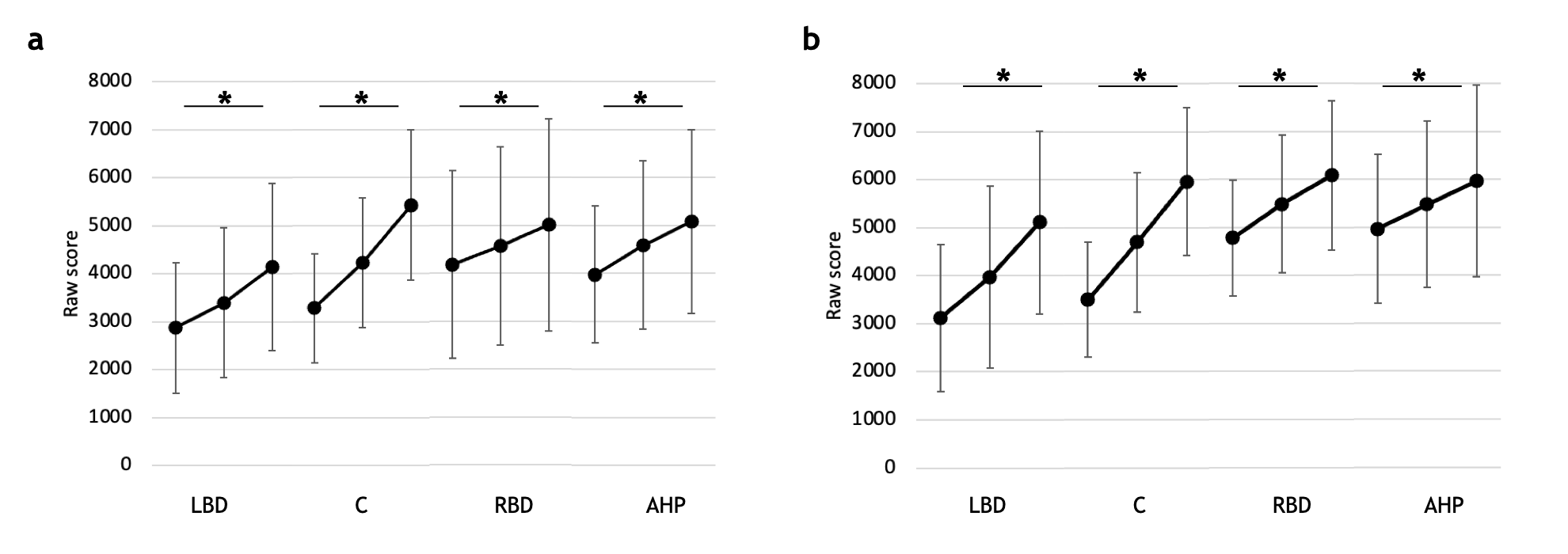
 **Figure SM1.** Mean and standard deviation of raw scores given by the patients for each duration, for each group in the a) ATE and b) MTE tasks. For each group, the circles represent the mean of the raw scores of 3000ms (left), 4500ms (centre), and 6500ms (right). The bars indicate standard deviations. * = p<0.01. LBD = left brain-damaged patients; C = controls; RBD = right brain-damaged patients; AHP = anosognosia for hemiplegia patients.

**Table SM1.** Post-hoc analysis of the model that investigated the differences in the patients’ raw estimates among the three durations of the ATE task (3000ms, 4500ms, 6500ms), for each group.

|  | Group | contrast | estimate | SE | df | z.ratio | p.value |
| --- | --- | --- | --- | --- | --- | --- | --- |
| ATE | AHP | 3000vs4500 | -489.694 | 135.1229 | Inf | -3.624 | 0.0008 |
|  |  | 3000vs6500 | -941.548 | 237.6846 | Inf | -3.961 | 0.0002 |
|  |  | 4500vs6500 | -451.854 | 148.9418 | Inf | -3.034 | 0.0068 |
|  | RBD | 3000vs4500 | -338.086 | 94.53603 | Inf | -3.576 | 0.001 |
|  |  | 3000vs6500 | -812.154 | 167.5656 | Inf | -4.847 | <.0001 |
|  |  | 4500vs6500 | -474.068 | 104.4235 | Inf | -4.54 | <.0001 |
|  | C | 3000vs4500 | -848.336 | 54.943 | Inf | -15.44 | <.0001 |
|  |  | 3000vs6500 | -2048.44 | 97.75154 | Inf | -20.956 | <.0001 |
|  |  | 4500vs6500 | -1200.1 | 60.83789 | Inf | -19.726 | <.0001 |
|  | LBD | 3000vs4500 | -347.328 | 101.7109 | Inf | -3.415 | 0.0018 |
|  |  | 3000vs6500 | -849.877 | 180.8503 | Inf | -4.699 | <.0001 |
|  |  | 4500vs6500 | -502.549 | 112.5671 | Inf | -4.464 | <.0001 |

**Table SM2.** Post-hoc analysis of the model that investigated the differences in the patients’ raw estimates among the three durations of the MTE task (3000ms, 4500ms, 6500ms), for each group.

|  | Group | contrast | estimate | SE | df | t.ratio | p.value |
| --- | --- | --- | --- | --- | --- | --- | --- |
| MTE | AHP | 3000vs4500 | -673.288 | 219.3394 | 70.45 | -3.07 | 0.0084 |
|  |  | 3000vs6500 | -1216.83 | 345.5205 | 69.63 | -3.522 | 0.0022 |
|  |  | 4500vs6500 | -543.544 | 233.106 | 70.51 | -2.332 | 0.0579 |
|  | RBD | 3000vs4500 | -751.94 | 143.2457 | 69.91 | -5.249 | <.0001 |
|  |  | 3000vs6500 | -1325.7 | 226.2531 | 69.68 | -5.859 | <.0001 |
|  |  | 4500vs6500 | -573.755 | 151.9705 | 69.38 | -3.775 | 0.001 |
|  | C | 3000vs4500 | -1164.83 | 83.30682 | 68.64 | -13.982 | <.0001 |
|  |  | 3000vs6500 | -2408.54 | 131.733 | 68.71 | -18.283 | <.0001 |
|  |  | 4500vs6500 | -1243.7 | 88.56293 | 68.67 | -14.043 | <.0001 |
|  | LBD | 3000vs4500 | -840.278 | 153.8962 | 68.49 | -5.46 | <.0001 |
|  |  | 3000vs6500 | -1889.45 | 243.7057 | 68.94 | -7.753 | <.0001 |
|  |  | 4500vs6500 | -1049.17 | 163.9256 | 69.03 | -6.4 | <.0001 |

**SM 3. Left- v. right hands differences**

In the ATE Hand task, a preliminary analysis investigated the differences in temporal estimation between left- and right-hand videos within each group. As no effect of the Effector condition emerged (Figure SM2), the errors regarding the left- and right-hand videos have been averaged for each subject.


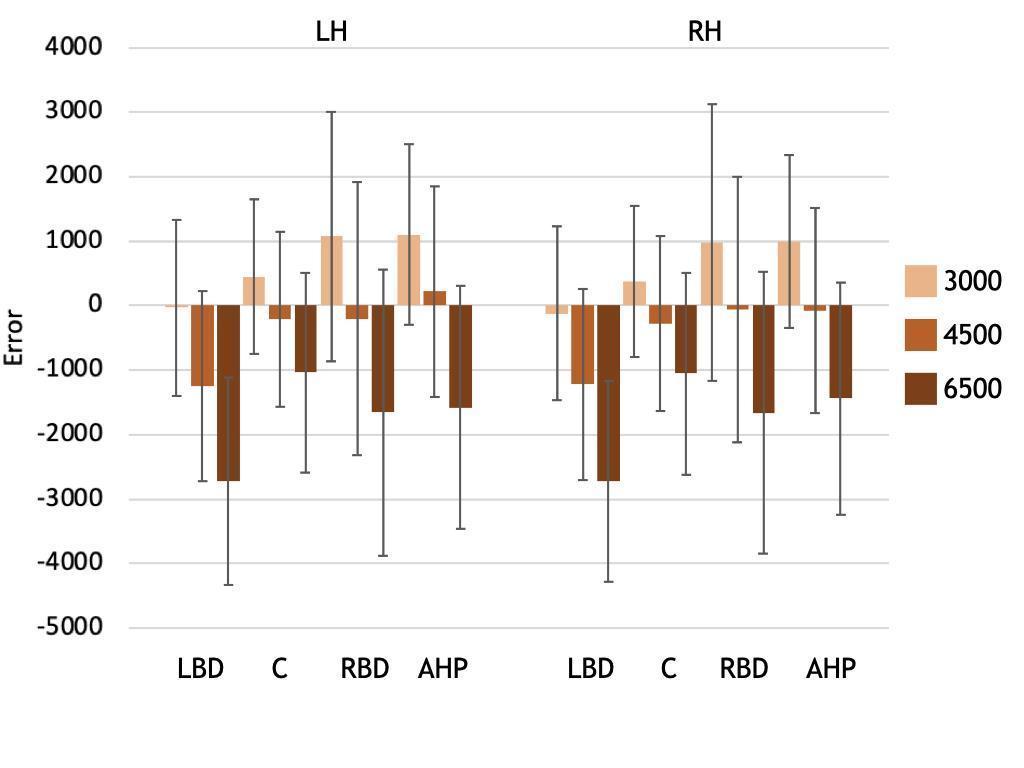


**Figure SM2.** Mean and standard deviation of errors divided for left (LH, left part of the graph) and right (RH, part of the graph) hands, for each duration, for each group in the ATE task. LBD = left brain-damaged patients; C = controls; RBD = right brain-damaged patients; AHP = anosognosia for hemiplegia patients.

**SM 3.** **ATE v. ATE-no Hand differences within the groups**

The within-group difference observed between ATE and ATE-No Hand tasks was found for the LBD group, which underestimate the duration of 6500ms in the ATE task compared to the ATE No Hand task (6500ms: t = -2.1; p = .04, Figure SM3).


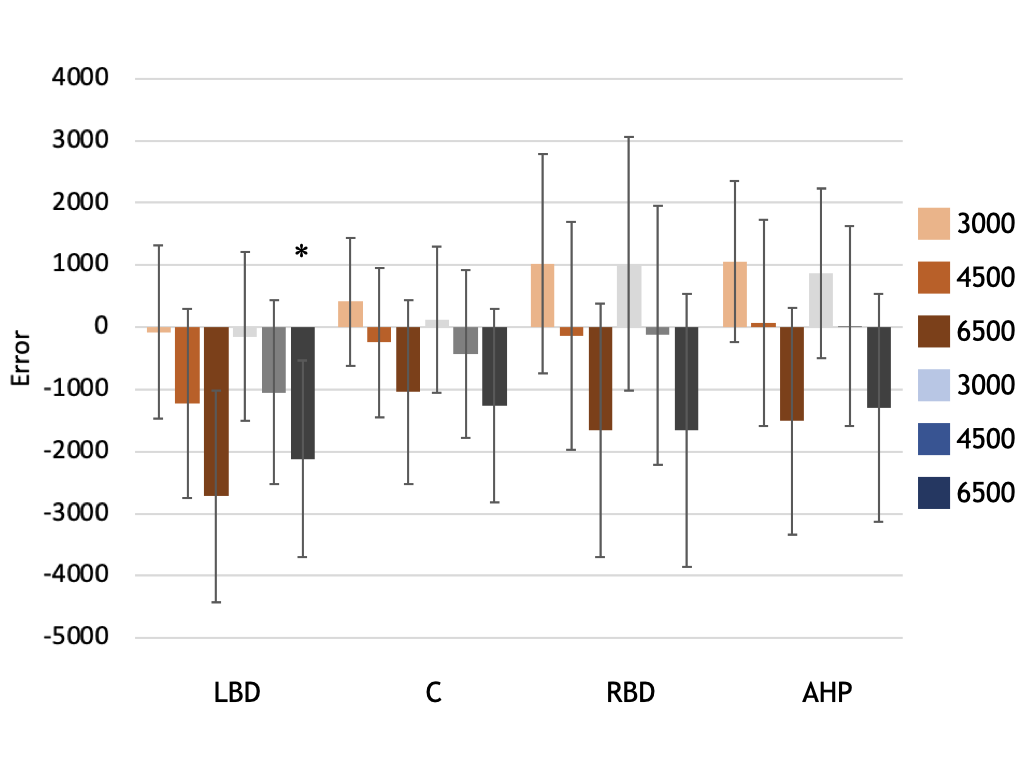


**Figure SM3.** The figure represents the mean and standard deviation of the ATE (in orange shades) and ATE-NH (in grey shades) for each group. * = p<.05. LBD = left brain-damaged patients; C = controls; RBD = right brain-damaged patients; AHP = anosognosia for hemiplegia patients.

**SM 4. Correlations with clinical variables**

**Table SM3.** Correlations of each task scores, divided for each duration, and demographic and clinical variables (z-score). adj p = Bonferroni corrected p values..

|  |  | 3000ms | | 4500ms | | 6500ms | |
| --- | --- | --- | --- | --- | --- | --- | --- |
|  |  | err (r) | err (adj p) | err (r) | err (adj p) | err (r) | err (adj p) |
| ATE   N = 33 | AAT token | 0.03 | 1 | 0.02 | 1 | 0.0 | 1 |
|  | AAT oral | -0.07 | 1 | -0.08 | 1 | -0.1 | 1 |
|  | Line Cros | -0.17 | 1 | -0.25 | 1 | -0.24 | 1 |
|  | Line Bis | -0.28 | 1 | -0.31 | 1 | -0.31 | 1 |
|  | VATA-m | 0.13 | 1 | 0.16 | 1 | 0.16 | 1 |
| MTE   N = 32 | AAT token | -0.05 | 1 | -0.1 | 1 | -0.17 | 1 |
|  | AAT oral | -0.06 | 1 | -0.02 | 1 | 0.03 | 1 |
|  | Line Cros | -0.18 | 1 | -0.18 | 1 | -0.15 | 1 |
|  | Line Bis | -0.41 | 1 | -0.32 | 1 | -0.23 | 1 |
|  | VATA-m | 0.19 | 1 | 0.17 | 1 | -0.06 | 1 |
| ATE-NH   N = 33 | AAT token | -0.07 | 1 | -0.02 | 1 | 0.05 | 1 |
|  | AAT oral | 0.12 | 1 | -0.12 | 1 | 0.08 | 1 |
|  | Line Cros | -0.08 | 1 | -0.08 | 1 | -0.08 | 1 |
|  | Line Bis | -0.25 | 1 | -0.24 | 1 | -0.18 | 1 |
|  | VATA-m | 0.16 | 1 | 0.19 | 1 | -0.16 | 1 |
| ATE-e   N = 16 | MMSE | 0.26 | 1 | 0.17 | 1 | 0.15 | 1 |
|  | Ideomotor | -0.31 | 1 | -0.25 | 1 | -0.23 | 1 |
|  | Line.Bis | -0.39 | 1 | -0.43 | 1 | -0.39 | 1 |
|  | VATA.m | -0.05 | 1 | 0 | 1 | 0.02 | 1 |

**SM 5. Results from the neuropsychological assessment of the participants in Experiment 2.**

**Table SM4.** Experiment 2. Mean and ± standard deviation of demographic, clinical and neuropsychological assessments are shown for the three groups of patients (RBD, AHP, LBD) and the healthy controls group. ^▲^Significantly differences of a group (AHP or LBD) with respect to the RBD group. ^■^ Significantly differences of a group (AHP or RBD) with respect to the LBD group. Only the MMSE was administered to participants in the control group, in order to exclude any signs of mental deterioration. Differences were found for the MMSE scores among the three groups (F = 12.39; df = 2; p = 0.001), particularly between the AHP and RBD (t=-4.96; df=13; p<0.001) and LBD groups (t=2.8; df=13; p=0.03) with worse performance in AHP. Significant differences were also found for the Line Bisection scores among the three groups (F = 9.7; df = 2; p = 0.002), particularly between the AHP and LBD groups (t=-4.41; df=13; p=0.002), with the AHP patients displaying more severe neglect for extrapersonal space than the LBD group. The Ideomotor apraxia scores were found to be different among the three groups (F = 5.56; df = 2; p = 0.018), with LBD showing more severe apraxia symptoms than the RBD patients (t=-3.33; df=13; p=0.014). Finally, as expected, a significant difference was confirmed for the VATA-m scores among the three groups (F = 39.68; df = 2; p<0.001) in particular between the AHP and RBD (t=8.84; df=13; p<0.001), and LBD groups (t=6.88; df=13; df=13; p<0.001).

|  | **RBD N = 7** | **AHP N = 4** | | **LBD N = 5** | | **Controls**  **N = 16** | |  |
| --- | --- | --- | --- | --- | --- | --- | --- | --- |
| **Age** | 64.57 ± 10.37 | | 66.25 ± 12.97 | | 70 ± 12.81 | | 61.94 ± 9.95 | |
| **Education** | 10.71 ± 5.53 | | 13 ± 4.08 | | 8.6 ± 4.93 | | 12.63 ± 4.33 | |
| **Interval (days)** | 74 ± 81.7 | | 53.5 ± 61.48 | | 66.2 ± 37.44 | |  | |
| **Motor index** (MRC-UUL) | 14.57 ± 19.57 | | 7.5 ± 15 | | 12.2 ± 19.41 | |  | |
| **Edinburgh Handedness Inventory** (EHI) | 83.57 ± 19.73 | | 82.5 ± 23.63 | | 85 ± 23.98 | | 78.75 ± 20.94 | |
| **Mini-Mental State Examination** (MMSE) | 25.92 ± 2.84 | | 20.63 ± 2.02^▲■^ | | 24.49 ± 2.88 | | 27.25 ± 1.33 | |
| **Token test (AAT)**  (number of errors) | 1.43 ± 2.57 | | 4.25 ± 0.96 | | 2.4 ± 2.88 | |  | |
| **Oral comprehension (AAT)** | 24.83 ± 4.88 | | 22 ± 1.73 | | 25.6 ± 4.62 | |  | |
| **Comprehension of written sentences (AAT)** | 23.5 ± 3.83 | | 22.67 ± 1.15 | | 24.2 ± 3.42 | |  | |
| **Ideomotor Apraxia**  (number of errors) | 0.14 ± 0.38 | | 0.25 ± 0.50 | | 1 ± 0.71^▲^ | |  | |
| **Digit span** (corrected scores) | 5.16 ± 0.79 | | 4.88 ± 0.70 | | 4.56 ± 1.13 | |  | |
| **Comb** | 0.03 ± 0.12 | | 0.12 ± 0.32 | | 0.01 ± 0.08 | |  | |
| **Line cancellation** | 33.43 ± 6.80 | | 21.75 ± 9.60 | | 35.6 ± 0.55 | |  | |
| **Line Bisection** | 6.43 ± 1.9 | | 2.75 ± 1.71^■^ | | 8.8 ± 0.45 | |  | |
| **VATA-m** (upper limb) | -2.14 ± 4.60 | | 16 ± 1.63^▲■^ | | -0.80 ± 1.92 | |  | |
| **Bisiach score** | 0 | | 1.38 ± 0.95 | | 0 | |  | |

**SM 6 The Graduation Scale used for participants’ responses**
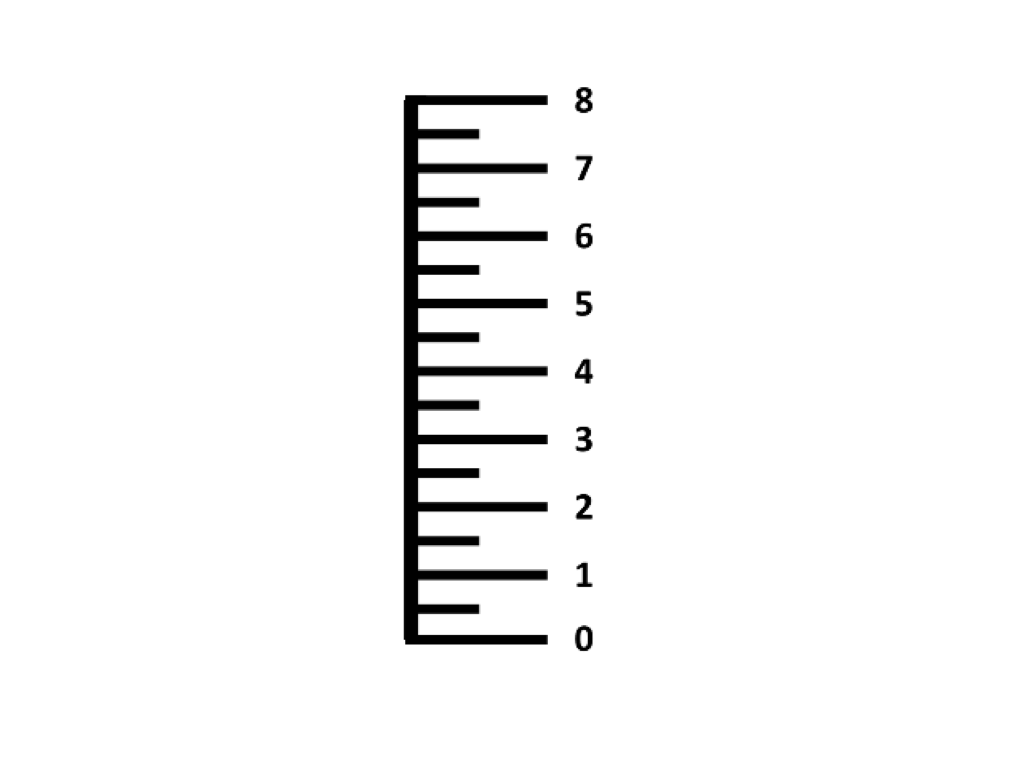


**Figure SM4.** Graduation scale. Throughout the four tasks, patients indicated their estimation of each action duration using the graduated scale here shown. The scale ranges from 0 to 8, with longer lines indicating the seconds, and shorter lines indicating the intervals of 0.5 seconds.

**SM 7 Comparison among the videos of the four tasks (ATE, MTE, NH and ATE-Embodiment)**

The videos of the four experimental tasks were produced in a way that maintains the greatest number of common characteristics. Stimulus and video durations were the same and when different durations were manipulated within a task (i.e. 3000 ms. 4500 ms. 6500 ms) these durations were the same in all tasks (the two tasks of experiment 1 and the two tasks of experiment 2). Similarly, the amplitude and velocity of the movements are similar (encoded frame per second: 60, for all the stimuli) and the movement direction is vertical. Finally, the absence of colours (black and white videos were used) harmonized other perceptive aspects.

To further analyse the potential effects of the differences in the stimuli, experimental findings were replicated by means of a model for each duration (lmer on R) with the interaction between the Group (each patients’ group and controls) and the Tasks as fixed effects, and Tasks as random effect. In this way, the results are controlled for the random effect linked to the task features. The results confirm that AHP and RBD groups significantly overestimate compared to controls the 3000 duration of the MTE (AHP: t-ratio 3; p-value (Tukey-corrected) = .003; RBD: t-ratio 4; p-value (Tukey-corrected) <.001) and NH (AHP: t-ratio 2.18, p-value (Tukey-corrected) .035; RBD: t-ratio 2.53; p-value (Tukey-corrected) = .015) tasks. Additionally, the new analysis confirms that the LBD group underestimate the 4500ms duration when compared to controls for the ATE (t-ratio 2.47; p-value(Tukey-corrected) = .017) task, as well as the 6500ms duration of the No Hand (t-ratio 2.2; p-value(Tukey-corrected) = .033) and the MTE (t-ratio 3.76; p-value; p-value(Tukey-corrected) =  <.001) tasks.


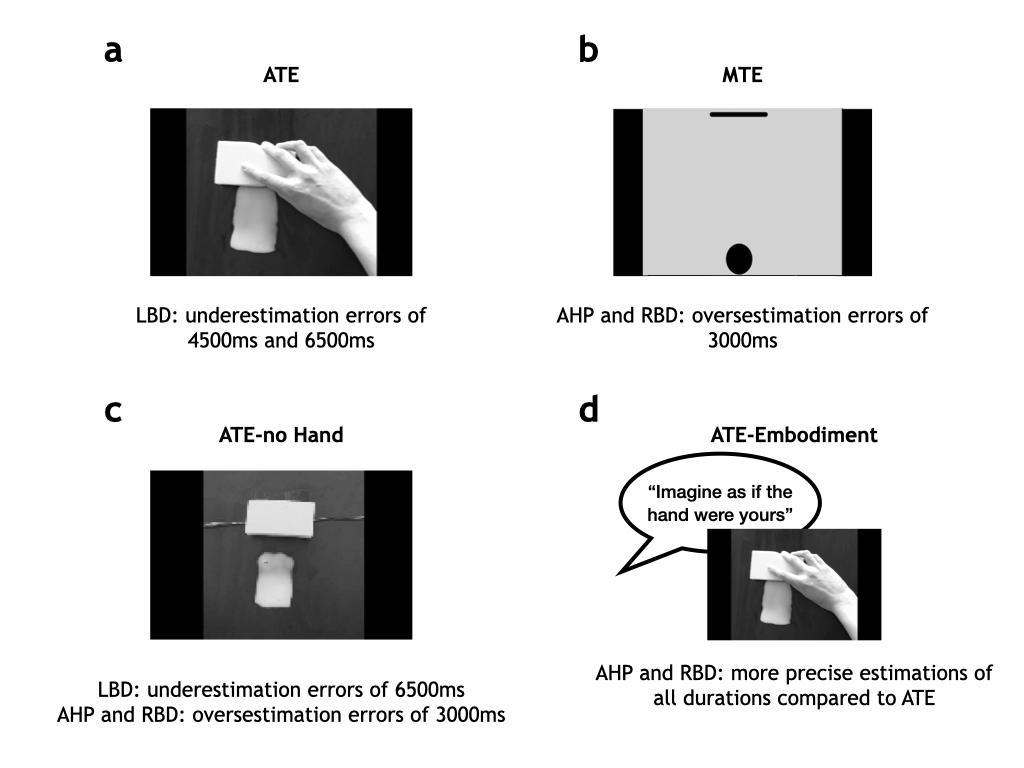


**Figure SM5.** Summary of the results for ATE, MTE, ATE-No Hand and ATE-Embodiment tasks. a) The comparison of the errors in ATE between each patients’ group and the controls indicates that the LBD group underestimate the duration of 4500 and 6500ms. b) For the MTE task, the comparison of the estimation errors between each patients’ group and the controls reveals that AHP and RBD patients overestimate the duration of 3000ms. c) In ATE-No Hand, the comparison of the error computed between each patients’ group and the controls shows that the LBD group underestimate the duration of 6500ms, while AHP and RBD groups overestimate the duration of 3000ms. d) The within-group comparison of the errors in the ATE and ATE-Embodiment task, computed for each duration and each patients’ group, reveals that AHP and RBD estimations are more precise in the ATE-Embodiment task. LBD = left brain-damaged patients; RBD = right brain-damaged patients; AHP = anosognosia for hemiplegia patients.
